# Medical Image Segmentation: A Comprehensive Review of Deep Learning-Based Methods

**DOI:** 10.3390/tomography11050052

**Published:** 2025-04-30

**Authors:** Yuxiao Gao, Yang Jiang, Yanhong Peng, Fujiang Yuan, Xinyue Zhang, Jianfeng Wang

**Affiliations:** 1 College of Artificial Intelligence, Taiyuan University of Technology, Jinzhong 036000, China; gaoyuxiao2550@link.tyut.edu.cn (Y.G.); zhangxinyue7136@link.tyut.edu.cn (X.Z.); 2 College of Mechanical Engineering, Chongqing University of Technology, Chongqing 400054, China; 3 School of Computer Science and Technology, Taiyuan Normal University, Taiyuan 030619, China; 4 School of Software, Taiyuan University of Technology, Jinzhong 036000, China

**Keywords:** deep learning, medical image segmentation, computer vision, CNNs, U-Net, transformer, GANs, SAM

## Abstract

Medical image segmentation is a critical application of computer vision in the analysis of medical images. Its primary objective is to isolate regions of interest in medical images from the background, thereby assisting clinicians in accurately identifying lesions, their sizes, locations, and their relationships with surrounding tissues. However, compared to natural images, medical images present unique challenges, such as low resolution, poor contrast, inconsistency, and scattered target regions. Furthermore, the accuracy and stability of segmentation results are subject to more stringent requirements. In recent years, with the widespread application of Convolutional Neural Networks (CNNs) in computer vision, deep learning-based methods for medical image segmentation have become a focal point of research. This paper categorizes, reviews, and summarizes the current representative methods and research status in the field of medical image segmentation. A comparative analysis of relevant experiments is presented, along with an introduction to commonly used public datasets, performance evaluation metrics, and loss functions in medical image segmentation. Finally, potential future research directions and development trends in this field are predicted and analyzed.

## 1. Introduction

With the rapid development and widespread adoption of imaging technologies in the medical field, medical image data have exhibited characteristics such as high growth rates, diverse categories, and significant real-world value, making them an essential resource in medicine. Given the large volume and complex structure of these data, the introduction of deep computing technologies has provided novel solutions for the efficient management, precise analysis, and extensive application of medical image data, playing a crucial role in disease diagnosis and treatment. Among these technologies, image segmentation, as a fundamental task in computer vision for medical image processing, divides image pixels into distinct regions, enabling the automatic localization and analysis of anatomical structures. This technology effectively extracts key regions in medical images, such as organs, blood vessels, and tumors, providing accurate and reliable data support for subsequent diagnosis, treatment, and research.

Early medical image segmentation algorithms primarily relied on traditional image processing techniques, including thresholding [1], edge detection [2], clustering [3], and graph theory-based methods [4]. While these approaches were simple to implement, they were prone to interference from imaging contrast, noise, lighting variations, and human factors, which limited their adaptability in complex images and constrained their segmentation accuracy and robustness. As research advanced, manually crafted feature-based algorithms became mainstream. These methods incorporated prior knowledge from medical experts to design features that reflect the characteristics of medical images, aiming to improve the accuracy of segmentation.

However, the design of these handcrafted features is heavily dependent on expert knowledge and suffers from poor generalization, making it difficult to transfer to new scenarios. Subsequently, automatic segmentation algorithms based on atlas templates emerged. These algorithms performed segmentation by registering standard atlas templates to medical images. However, constructing these atlas templates is typically time-consuming, and anatomical differences between individuals, as well as morphological changes caused by diseases, may introduce registration errors, thereby affecting segmentation performance. With the growing volume and diversity of medical image data, these methods gradually revealed their limitations in handling the complexities and variability of real-world situations, failing to produce optimal segmentation results.

In recent years, with the development of deep learning technologies and the growth of telemedicine, Convolutional Neural Networks (CNNs) have made significant advancements in the field of medical image segmentation. CNNs can automatically learn features from medical images and accurately segment different tissues or lesion regions. Compared to traditional algorithms, deep learning-based medical image segmentation techniques have been widely applied in clinical research and auxiliary diagnosis due to their powerful feature extraction and generalization capabilities. Furthermore, deep learning algorithms are capable of fully exploiting the rich information and deep features within medical image data, providing robust technical support for early disease diagnosis, precise localization, and treatment monitoring. For example, in applications such as tumor detection, angiography, and brain tissue analysis, deep learning techniques can achieve high-accuracy segmentation of target regions, assisting clinicians in more detailed and comprehensive disease assessment and treatment planning. Despite the enormous potential of deep learning in medical image segmentation, medical images possess inherent characteristics that distinguish them from natural images, including the following:

(1) Diversity of Medical Image Modalities [5]:

Medical images typically consist of multiple modalities, which differentiates them from natural images that generally have a single modality. For example, in the diagnosis of cardiovascular diseases [6], commonly used medical imaging modalities include echocardiography, cardiac magnetic resonance imaging (CMR), and cardiac computed tomography angiography (CTA). Consequently, in cardiac image segmentation tasks, it is often necessary to integrate information from different modalities to improve the accuracy of segmentation.

(2) Blurred Edges in Medical Images [7]:

Due to limitations in imaging technologies, human factors, image processing equipment, and parameters, medical images often suffer from issues such as noise, unclear boundaries, low resolution, and insufficient contrast. These challenges complicate the identification of lesions, feature analysis, and treatment planning, potentially leading to missed or misdiagnosed lesions by clinicians, which in turn can affect diagnostic accuracy and treatment outcomes.

(3) Scarcity of Annotated Medical Image Data [8]:

Obtaining medical image data is inherently difficult, especially in the case of rare disease cases. Annotating medical images requires significant time and effort and demands annotators to have deep medical knowledge, such as an understanding of human anatomy and disease characteristics. As a result, annotated medical image data are scarce, posing a considerable challenge for training deep learning models and necessitating a reduction in reliance on precise pixel-level annotations.

(4) Complex and Diverse Segmentation Targets in Medical Images [9]:

The segmentation targets in medical images, such as organs, tissues, or lesions, exhibit complex and irregular shapes. For example, the intricate networks of bronchi and blood vessels in the lungs, or the morphological variations of tumors, contribute to the diversity and complexity of segmentation targets. These shape variations add significant challenges to medical image segmentation tasks, especially when dealing with lesions like tumors, where the boundary between the tumor and surrounding tissues is often ambiguous and may involve mutual infiltration.

**Figure 1:**
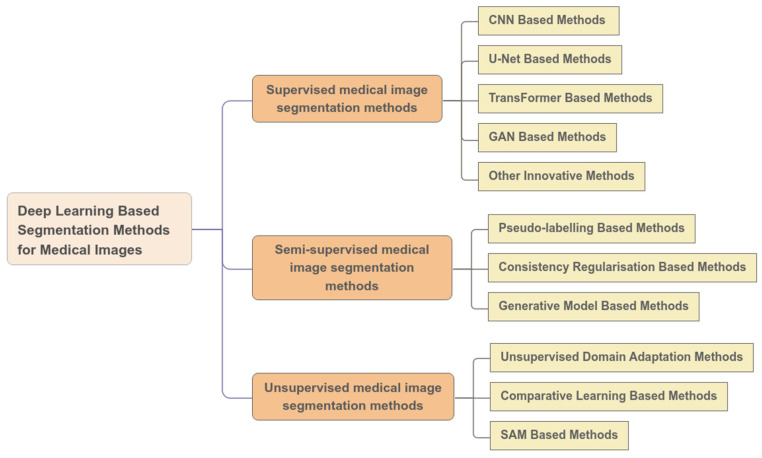
Classification of deep learning-based methods for medical image segmentation.

In summary, deep learning holds great promise for medical image segmentation, but it also faces numerous challenges that need to be addressed. To investigate the application of deep learning in this field, this study employs a qualitative research methodology. Using Google Scholar, we conducted searches with the keywords “medical image segmentation” combined with each of the terms “deep learning”, “supervised learning”, “semi-supervised learning”, “unsupervised learning”, “CNNs”, “GANs”, “transformer”, “U-Net”, and “SAM”. As shown in Figure 1, this paper provides a summary of the currently representative deep learning-based medical image segmentation methods, classifying them into three categories based on the learning approach: supervised learning, semi-supervised learning, and unsupervised learning. For each category, the paper analyzes the representative algorithms, detailing the fundamental concepts, advantages, limitations, and application scenarios of each method. Furthermore, it systematically discusses the contributions of deep learning to medical image segmentation. Since some methods may incorporate multiple technical concepts, the classification presented in this paper is intended to facilitate systematic discussion rather than strictly mutually exclusive categorization.

## 2. Supervised Learning Algorithms for Medical Image Segmentation

**Figure 2:**
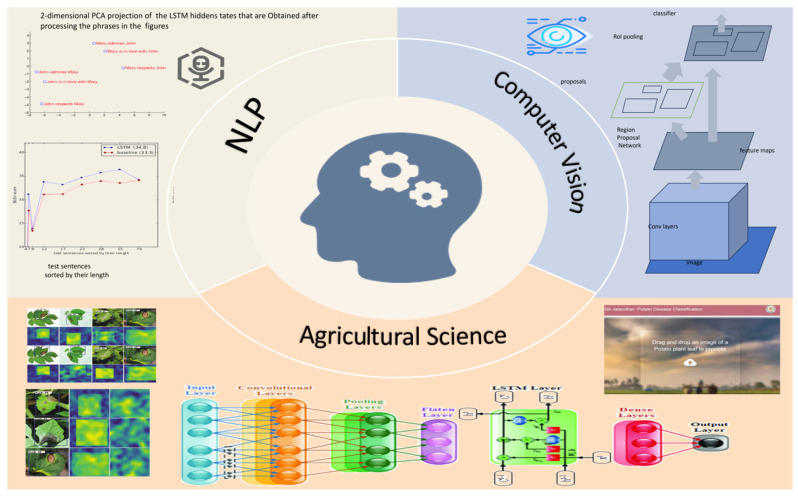
Application scenarios for deep learning [14,17].

The concept of deep learning, introduced by Hinton et al. [10], is a machine learning technique based on artificial neural networks. By constructing multi-layer neural network models, deep learning enables computers to automatically learn features and patterns from large datasets to perform tasks such as classification, prediction, and segmentation [11]. As shown in Figure 2, due to its powerful feature learning and nonlinear modeling capabilities, deep learning has been widely applied in various fields, including computer vision [12,13], natural language processing [14,15,16], and agricultural sciences [17,18,19,20]. Moreover, cognitive computing and soft computing techniques have shown effectiveness in predicting complex fluid systems, which may offer insights for modeling non-rigid anatomical structures in medical image segmentation [21,22]. Additionally, deep learning can efficiently perform image segmentation by learning the feature representations of images and automatically extracting semantic and spatial information. In this context, deep learning has rapidly emerged in the field of medical image segmentation and become an essential tool in clinical medical diagnostics. Initially, deep learning-based medical image segmentation predominantly employed supervised learning methods, relying on large amounts of accurately annotated medical image data for training. This enables the computer to learn the characteristics of different tissues, organs, and lesion regions, thereby achieving precise segmentation of medical images and significantly improving the efficiency and accuracy of medical diagnostics. This section will introduce the typical supervised learning algorithms in the field of medical image segmentation.

### 2.1. CNN-Based Methods

**Figure 3:**
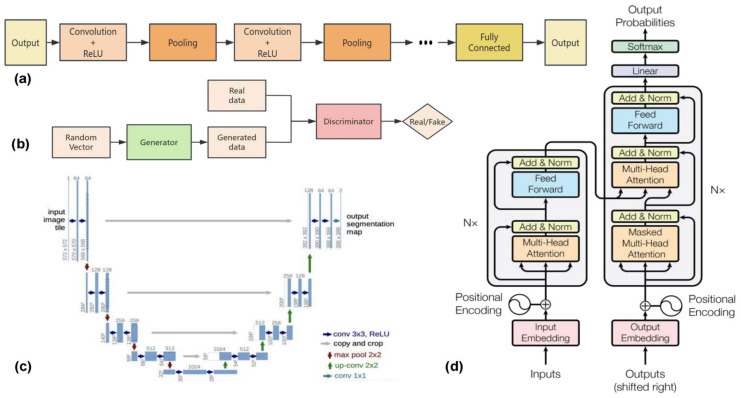
(**a**) The CNN model architecture; (**b**) The GAN model architecture; (**c**) The U-Net model architecture [25]; (**d**) The TransFormer-model architecture [16].

Convolutional Neural Networks (CNNs), initially proposed by Fukushima et al. [23], are widely used deep learning models for processing grid-structured data, such as images. As shown in Figure 3a, CNNs consist of several key layers, including the convolutional layer, pooling layer, and fully connected layer. The convolutional layer extracts local features by applying convolutional kernels over the input data. The pooling layer reduces the spatial dimensions of the data, which helps to decrease computational complexity while retaining important features. CNNs have become a cornerstone in computer vision [24] and have shown significant potential in medical image segmentation. In recent years, numerous CNN-based approaches have been developed to address challenges in medical imaging, improving the accuracy of segmentation and efficiency. The following sections will review the most prominent CNN-based methods and their applications in medical image segmentation.

#### 2.1.1. Colonoscopy Image Processing Methods

Colorectal cancer (CRC) is the third most common cancer globally and the second leading cause of cancer-related death, following lung cancer. According to statistics [26], approximately 1.94 million new cases of CRC and 900,000 deaths occurred worldwide in 2022, accounting for 9.6% and 9.3% of all cancer cases, respectively. Colorectal polyps are lesions on the intestinal mucosa, which, if not intervened in in a timely manner, may develop into malignant tumors within 10 to 15 years [27]. Therefore, early detection and treatment of polyps are crucial in reducing the incidence and mortality rates of patients. Polyp segmentation provides detailed morphological information, which is important for assessing the malignancy potential of polyps and formulating personalized treatment plans.

To address this, Cai et al. [28] proposed a perspective-aware network framework, VANet (Vanishing Attention Network), for polyp detection and segmentation in colonoscopy images. By treating polyps as discriminative features, VANet enhances the network’s ability to perceive polyp boundaries. Compared to other algorithms, the Dice coefficient was improved by approximately 6%, and it effectively handles colonoscopy images in real-world scenarios. However, issues such as variations in lighting, inconsistent texture and shape, and color distribution inconsistencies in colonoscopy images still limit VANet’s performance in boundary segmentation and make it challenging to distinguish polyps from surrounding tissues, leading to misclassification. To overcome these issues, Du et al. [29] introduced the ICGNet, a reverse contour-guided network based on integrated context. ICGNet incorporates three lightweight modules: the Reverse-Contour Guidance Module (RCG), the Adaptive Local–Global Context Module (ALGM), and the Hybrid Pyramid Pooling Fusion Module (HPPF), which effectively enhance feature representation and multi-scale information fusion, significantly improving segmentation performance. Despite addressing boundary blurring and detail loss, as well as improving detection accuracy and robustness, ICGNet neglects the inconsistency in image color distribution, leading to overfitting.

Furthermore, a reliable segmentation model should not only provide high-accuracy results but also incorporate uncertainty measurement methods to assist clinicians in making informed decisions. In this regard, UM-Net [30] improves upon ICGNet by introducing a color transfer operation that weakens the relationship between color and polyps, thereby allowing the model to focus more on the shape of the polyps. However, UM-Net still requires further refinement in its model design and training strategies to enhance robustness and accuracy in more complex scenarios, such as variations in background brightness, especially in challenging medical image environments.

To address the poor performance of traditional algorithms when handling polyps of varying sizes and shapes, Zhang et al. [31] proposed ACSNet (Adaptive Context Selection Network), which utilizes an adaptive context selection framework to effectively leverage both local and global contextual information. This approach significantly improves sensitivity to size variation in colon polyps, achieving a Dice coefficient of 91.3% on the Kvasir-SEG dataset. However, its ability to capture fine-grained edge features of polyps is still limited by the local context modeling capabilities. To overcome this, PraNet [32](Parallel Reverse Attention Network) innovatively introduced a reverse attention mechanism (RA) that progressively refines edge details from high-dimensional semantic features, excelling in handling low-contrast polyps. SANet [33] (Shallow Attention Network) further enhances segmentation quality by filtering out background noise from shallow features through a shallow attention module, thereby improving the model’s performance in segmenting small polyps.

Traditional segmentation methods struggle with highly deformable anatomical structures. Recent approaches have incorporated bio-inspired techniques to better model these complex morphologies. For example, circular soft actuators [34] have been explored for simulating the defecation process in the human rectum, providing insights into modeling physiological deformations in medical imaging.

To visually demonstrate the performance differences of the aforementioned polyp segmentation methods in practical applications, this study collected experimental data from these methods on the Kvasir-SEG and EndoScene medical image datasets. A series of scientific evaluation metrics were employed to quantitatively analyze each method, with detailed results presented in Table 1. The results reveal notable differences in the performance of various polyp segmentation methods across the Kvasir-SEG and EndoScene datasets. UMNet consistently outperforms other methods, achieving the highest Dice scores (93.04% on Kvasir-SEG and 89.26% on EndoScene) and the lowest mean absolute error (MAE) values (2.31 and 1.38, respectively), indicating superior accuracy of segmentation and minimal boundary errors. ICGNet and SANet also demonstrate strong performance, with competitive Dice scores and relatively low MAE values, making them viable alternatives for medical image segmentation tasks. Moreover, methods such as UNet and U-Net++ show comparatively lower performance, particularly in terms of Dice scores and MAE, suggesting limitations in capturing complex polyp structures.

#### 2.1.2. Coronary Artery Segmentation Methods

Coronary artery disease is the most common type of cardiovascular disease, primarily caused by the narrowing or blockage of the coronary arteries, leading to myocardial ischemia or even necrosis. Accurately extracting the coronary artery tree is a crucial step in diagnosing coronary artery disease. Traditional machine learning-based segmentation methods [30] rely on manually designed features, which struggle to comprehensively and accurately represent the complex characteristics of coronary arteries.

**Figure 4:**
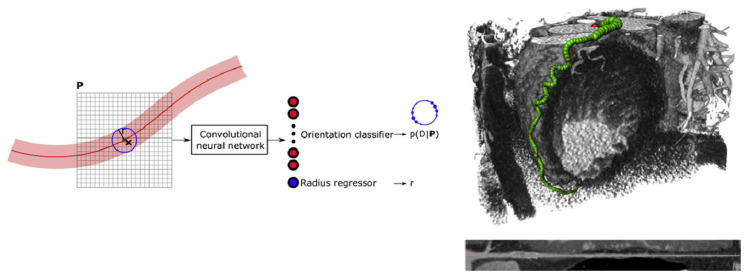
CNN-based method for coronary artery centerline extraction [35].Centerline extraction was successfully achieved in a patient with tortuous coronary arteries. The spheres represent centerline points, with their radii corresponding to automatically determined radius values. The colors of the spheres indicate the uncertainty of the classifier. Green signifies low entropy values, while red (at the ostium and the terminal end of the centerline) represents high entropy.

To address this issue, Wolterink et al. [35] proposed a convolutional neural network (CNN)-based method for coronary artery centerline extraction. As shown in Figure 4, this approach requires only a single manually placed seed point to track the entire vascular tree, enabling fully automated extraction of the coronary artery tree. This method allows for rapid and accurate coronary artery extraction in large-scale CCTA scans. Similarly, Shahzad et al. [36] introduced an automated method for detecting, quantifying, and segmenting the coronary arteries. This approach automates vessel segmentation and stenosis detection, significantly reducing the workload and error rate for physicians. Kong et al. [37] proposed a novel tree-structured convolutional gated recurrent unit (ConvGRU) model to learn the anatomical structure of the coronary arteries, improving both the accuracy and efficiency of segmentation.

However, traditional segmentation methods typically require the extraction of the coronary artery centerline before performing lumen segmentation. This approach is prone to errors, which can lead to inaccurate segmentation results. To address this issue, Zreik et al. [38] proposed a recursive convolutional neural network (CNN) method for the automatic detection and classification of coronary artery plaques and stenosis, which only requires the input of the coronary artery centerline and CCTA images to complete the task. However, in practical applications, arteries and veins often have similar intensities and are in close proximity, making them prone to confusion. To overcome this, Wang et al. [39] introduced the AVDNet network, which simultaneously performs coronary artery and vein segmentation. By including both the coronary artery and vein in the segmentation task, this method improves detection accuracy and reduces false predictions. However, this approach is currently only applicable to the segmentation of coronary arteries and veins, and its effectiveness for segmenting other types of vessels remains to be further validated.

With the advancement of deep learning technology, significant progress has been made in the field of vessel segmentation, but there are still many challenges in practical applications. The blurred details and noise interference in low-quality images, as well as the complex and variable morphology of blood vessels and their intricate branches, continue to present significant challenges for existing algorithms. Future research could address these challenges from multiple aspects: on one hand, algorithms need to be enhanced in terms of robustness and generalization to handle vessel segmentation tasks in low-quality images and complex vascular structures; on the other hand, the scope of data utilization could be expanded by integrating clinical information, electrocardiograms, magnetic resonance imaging, and other imaging data sources, thus providing more comprehensive information to support vessel segmentation and improving the accuracy of segmentation.

#### 2.1.3. Interactive Medical Image Segmentation Methods

**Figure 5:**
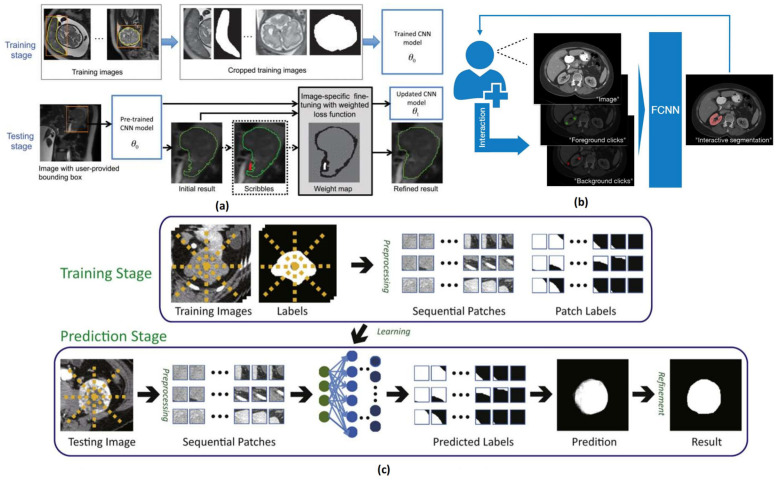
(**a**) A bounding box-based framework for interactive image segmentation [42]. (**b**) Schematic of the semi-automatic segmentation method based on user clicks [44]. The user observes the image and clicks on the region of interest (green Gaussian); a 2D segmentation result is generated almost instantly. (**c**) The point-based approach to interactive medical image segmentation [45].

In traditional image segmentation, models perform segmentation solely based on the input images without considering the specific needs and contexts of the user. To address this limitation, interactive image segmentation emerged [40], allowing users to interact with the model to achieve more precise and flexible segmentation results. Deep learning and convolutional neural networks (CNNs) have achieved state-of-the-art performance in interactive medical image segmentation [41]. However, when using CNNs for interactive segmentation, their limited generalization ability presents a challenge, as current CNN models cannot effectively handle object categories that were not present in the training set. To overcome this, Wang et al. [42] proposed a boundary box-based interactive segmentation framework. As shown in Figure 5a, this method enables interactive segmentation using boundary boxes and user-provided scribbles. Experimental results demonstrate that the model can accurately recognize previously unseen objects while requiring less user interaction and time. Similarly, Wang et al. [43] designed an end-to-end interactive segmentation network, combining user-provided initial scribbles with CNNs to improve the accuracy and robustness of automatic segmentation results, thereby reducing user intervention and time costs.

Sakinis et al. introduced a semi-automatic segmentation method based on user clicks, as shown in Figure 5b. With this method, users can simply click on any structure they wish to segment, allowing for real-time interaction with the segmentation results, achieving faster user interaction compared to scribble-based methods. Similarly, Luo et al. [46] proposed an innovative interactive segmentation approach that not only requires clicks as user input but also generalizes well to a range of previously unseen objects. Building on these ideas, Zhang et al. [45] introduced a point-based interactive medical image segmentation method, as shown in Figure 5c. In this approach, users only need to click on the rough center of an object prior to segmentation, enabling high-performance segmentation while reducing segmentation time.

The methods discussed above primarily focus on segmentation tasks involving individual organs or objects in medical images. However, these approaches have certain limitations when applied to complex scenarios involving multiple targets of different categories. To address this issue, Kaushal et al. [47] incorporated Swarm Intelligence (SI) alongside CNNs for image segmentation to optimize the identification of relevant regions. Their method achieved a segmentation accuracy of 96.45%, with an average processing time of 9.09 s. However, custom interactive segmentation solutions require a separate model for each task, resulting in redundant training times and parameters [48]. To overcome this, Ding et al. [49] proposed a unified framework, S2VNet, to tackle both automatic and interactive medical image segmentation tasks. Experimental results on the WORD dataset showed that this method outperformed others in both the automatic and interactive modes. However, since S2VNet is based on CNNs, it fails to capture global features, and its performance tends to degrade on larger datasets. Future work could involve increasing the number of CNN layers to enhance the model’s learning capacity.

With the application of CNNs in interactive medical image segmentation, significant progress has been made in the field [50]. Additionally, CNNs have shown potential in integrating multi-modal sensory data, including wearable haptic feedback devices designed for kinesthetic perception [51,52]. Such integration could enhance real-time medical applications, particularly in rehabilitation and prosthetic control. Nevertheless, in practical scenarios, interactive medical image segmentation still faces several challenges, including inaccuracies in segmenting multi-class targets in complex scenes and difficulty capturing global features. Future research can explore multiple avenues: on one hand, enhancing the algorithm’s ability to handle complex scenes and multi-class targets while improving model generalization; on the other hand, combining other technologies to address CNNs’ limitations in capturing global features, thereby improving the accuracy of interactive medical image segmentation.

### 2.2. U-Net-Based Algorithms

As a classic convolutional neural network architecture, U-Net [25] has achieved outstanding results in medical image segmentation tasks since its inception. As shown in Figure 3c, its unique encoder–decoder structure and skip connection mechanism effectively address the problem of feature loss, enabling the model to maintain excellent segmentation performance even with limited training data. However, with the increasing complexity of medical image data and the growing demands for the accuracy of segmentation, researchers have made numerous improvements and innovations based on U-Net, leading to the development of a series of advanced algorithms.

Traditional U-Net treats all features equally during feature fusion, failing to emphasize the importance of key features, which may result in interference from irrelevant features. To address this issue, Attention U-Net [53] introduced an attention module into U-Net’s skip connection part, allowing it to adaptively adjust the weight of feature maps. Experimental results on the CT-82 and CT-150 datasets showed significant improvements in the Dice coefficient, enabling more precise delineation of the target region’s boundaries. While U-Net’s skip connection mechanism retains feature information to some extent, it still has limitations in deeper feature fusion. To overcome this, UNet++ [54] introduced dense skip connections, enabling the network to more effectively transmit and fuse multi-scale features, thus improving segmentation accuracy. Additionally, to handle 3D medical imaging data more efficiently, Cicek et al. [55] proposed 3D U-Net, extending U-Net’s 2D operations into the three-dimensional space. This extension significantly enhanced the efficiency and accuracy of 3D medical image segmentation, making it a crucial tool in the field of medical image analysis.

Medical images contain rich multi-scale information and complex interactions between features at different levels. However, traditional U-Net often fails to produce satisfactory segmentation results for lesions with blurry boundaries and irregular shapes. To address this, Dai et al. [56] proposed a dual-path U-Net architecture, I^2^U-Net. Through deep information exchange between the two paths, I^2^U-Net enables the reuse and exploration of historical information, allowing the deep network to learn more comprehensive features. Compared to other state-of-the-art methods, the average Dice coefficient improved by approximately 3%. Similarly, R2U-Net [57] introduced a recurrent structure and residual modules to the U-Net architecture, better adapting to the characteristics of complex medical image data. This approach demonstrated excellent performance in tasks such as liver and kidney segmentation, significantly enhancing segmentation accuracy and model robustness.

Traditional medical image segmentation algorithms require manual parameter tuning or the design of new network architectures to adapt to different datasets and tasks, which is time-consuming and susceptible to human bias. To address this, Isensee et al. [58] proposed nnU-Net, a medical image segmentation framework. nnU-Net has powerful automation and standardization capabilities, automatically analyzing dataset characteristics and selecting the appropriate network architecture, preprocessing methods, and training strategies. It can be rapidly deployed in various medical image segmentation tasks, achieving good results. However, its performance may not match that of manually tuned methods in certain specialized datasets or complex tasks, and the automation process offers limited interpretability.

### 2.3. Transformer-Based Methods

In medical imaging, target structures often exhibit complex spatial relationships with surrounding tissues, requiring the capture of long-range contextual information for accurate segmentation. Traditional CNNs primarily extract features through local convolutional operations, which are limited in their ability to capture long-range dependencies. To overcome this limitation, the Transformer architecture [16] (Figure 3d), originally designed for natural language processing [59], leverages a powerful self-attention mechanism to capture global dependencies across long distances in the input data. Unlike CNNs, which apply fixed receptive fields, Transformers allow for dynamic learning of relationships between distant pixels, making them particularly well-suited for tasks requiring long-range contextual understanding, such as medical image segmentation.

A Transformer model consists of an encoder–decoder architecture, with the encoder focusing on extracting features from input sequences and the decoder reconstructing the output. The core of the Transformer is the self-attention mechanism, which computes the relationships between all pairs of positions in the input sequence, assigning weights to different parts of the data based on their relevance to the task at hand. Additionally, components such as positional encoding are used to inject spatial information into the model, addressing the lack of inherent sequence ordering in the raw data. Layer normalization and multi-head attention further enhance the model’s ability to capture complex dependencies and mitigate issues like vanishing gradients. In recent years, researchers have begun to incorporate Transformers into medical image segmentation tasks, leading to the development of a series of innovative algorithms that have significantly advanced the field.

Chen et al. [60] innovatively combined the Transformer with the classic U-Net architecture to develop the TransUNet model, achieving effective integration of global and local features. In multi-organ segmentation tasks on the Synapse dataset, TransUNet outperformed other methods, improving the Dice coefficient by 1.5% to 11.2%, significantly enhancing the accuracy of segmentation and robustness. However, the integration of the Transformer architecture with traditional CNNs has limitations, as it struggles to effectively capture the overall features of targets in medical imaging. To address these issues, the SETR [61] model was proposed. SETR is a pure Transformer-based medical image segmentation network that directly inputs medical images as sequences into the Transformer encoder, leveraging the Transformer’s global modeling capabilities. However, SETR cannot directly handle large-scale image data due to excessive computational costs, and it struggles to capture multi-scale information, making it unsuitable for images with varying resolutions.

To overcome these limitations, several improvements have been proposed. UNetr [62] innovatively combines a pyramid-like multi-scale feature extraction mechanism with global context modeling, significantly enhancing the model’s ability to process images at different scales. However, Transformer architectures are typically larger and more complex than traditional convolutional neural networks, requiring substantial computational resources and training time. Building upon this, Xie et al. [63] introduced CoTr, which utilizes a lightweight Transformer module paired with a sparse attention mechanism, greatly reducing the computational resources and training time required for large-scale medical image data while still capturing global contextual information. Experimental results show that CoTr improved the Dice coefficient by approximately 3% on the BCV dataset, significantly outperforming previous methods.

In addition to sparse attention, axial Transformer architectures have also been explored as an effective solution to address the computational challenges in 3D medical image segmentation. Axial Transformers, by applying attention only along individual axes (height, width, and depth), reduce the computational complexity significantly when processing 3D data. This approach is particularly beneficial in medical image segmentation tasks, where high-dimensional data (such as 3D MRI or CT images) need to be processed efficiently. For instance, the work by Du et al. [64] introduced Axial-DeepLab, which demonstrated significant improvements in computational efficiency and the accuracy of segmentation, particularly in 3D image segmentation tasks. By focusing on each axis independently, axial Transformers effectively capture the local dependencies of the data while minimizing computational costs, making them a promising direction for future research in medical image analysis.

Traditional CNNs often face a trade-off between computational efficiency and model capacity when processing large-scale medical imaging data [64]. To address this issue, Cao et al. [65] proposed Swin-UNet, which integrates features from different levels to enhance segmentation capabilities for objects of varying sizes and shapes. This model maintains the global modeling capabilities of the Transformer while significantly improving computational efficiency. However, the design based on 2D image slices struggles to capture the voxel-level spatial continuity in 3D medical images. To overcome this limitation, TransBTS [66] was the first to combine 3D CNNs with Transformers, achieving three-dimensional segmentation of brain tumors through local–global feature collaboration. On the BraTs2019 dataset, TransBTS achieved Dice coefficients of 78.93%, 90.00%, and 81.94% for ET, WT, and TC, respectively, providing a novel and efficient solution for medical image segmentation. Similarly, VSmTrans [67] is a new Transformer structure for 3D medical image segmentation tasks. This method leverages the natural and built-in inductive advantages of convolutions, addressing issues such as the difficulty traditional CNNs face in capturing global information and the high pre-training data requirements. However, when handling very small datasets, its performance does not outperform pure convolutional architectures. Future research should explore how to improve the performance of VSmTrans on small datasets.

To address issues such as insufficient positional dependency and inadequate local feature extraction in traditional models, Chu et al. [68] proposed a medical image segmentation model based on a dual-coordinate attention mechanism, DCCAT, which enables automatic segmentation of thrombus in coronary optical coherence tomography (OCT) images. Compared to single-coordinate image input, dual-coordinate input significantly enhances model performance, especially in thrombus and guidewire detection. In scenarios with limited data, the performance of the DCCAT model surpasses that of traditional CNN and Transformer models, with a notable improvement of approximately 5% in the Dice Similarity Coefficient (DSC). However, this method requires substantial training data and pre-processing of input images, which increases computational complexity. Future research could focus on reducing the dependence on large training datasets while improving the model’s robustness and generalization ability. Additionally, exploring integrations with other techniques, such as transfer learning and meta-learning, may further enhance performance.

The previous section provided a comprehensive overview of Transformer- and U-Net-based models in the field of medical image segmentation. U-Net, with its classic U-shaped architecture, leverages convolution, pooling, upsampling, and feature fusion operations to balance local and global information, providing the foundational paradigm for medical image segmentation and serving as the starting point for many model improvements. Transformer, with its powerful global modeling capacity and self-attention mechanism, captures long-range dependencies, offering new perspectives and methods for medical image segmentation. These models play a crucial role in medical image segmentation, although their performance in practical applications varies. To clarify the differences in performance of these Transformer- and U-Net-based models in real-world applications, this study presents a quantitative comparison of various typical methods across different medical image datasets, with detailed results shown in Table 2 and Table 3.

In Table 2, the standard U-Net demonstrated moderate performance across different datasets, with Dice scores ranging from 82.31% (Kvasir) to 89.28% (ClinicDB). However, its performance on the EndoScene dataset was relatively lower, indicating potential limitations in handling complex polyp structures. The improved U-Net variants (AttU-Net, U-Net++, nnU-Net) introduced certain optimizations based on the original U-Net, but they did not consistently outperform the standard U-Net across all datasets. Meanwhile, Transformer-based models, Trans-Unet and Swin-Unet, demonstrated strong segmentation capabilities. Notably, Swin-Unet achieved the highest Dice score (87.50%) on the Kvasir dataset, outperforming nnU-Net and U-Net++. This suggests that Transformer models, leveraging their global attention mechanisms, effectively capture long-range dependencies, thereby improving segmentation accuracy in complex medical images.

In Table 3, UNETR achieved the highest Dice score and the lowest HD95, outperforming U-Net and Transformer-based models. Its effective integration of Transformer features with CNN-based local processing led to the higher accuracy of segmentation. For brain tumor segmentation, UNETR again outperformed other models in whole tumor segmentation, demonstrating strong generalization ability. However, in enhanced tumor and tumor core (TC) segmentation, TransBTS achieved the best Dice score for ET and the lowest HD95 for TC, highlighting its advantage in segmenting more challenging tumor subregions. Overall, while U-Net remains a strong baseline, Transformer-based models show significant advantages in handling complex structures like polyps and brain tumors. Given the varying performance across tasks and datasets, hybrid approaches combining CNN and Transformer features may offer the most effective solution for medical image segmentation.

### 2.4. GAN-Based Methods

In the field of medical image segmentation, traditional methods face challenges in accurately capturing complex features and addressing data imbalance issues. The former limits segmentation accuracy, particularly in cases with irregular tumor boundaries or complex internal structures, while the latter leads to poor segmentation performance for minority-class pixels. Generative Adversarial Networks (GANs), with their unique architecture and training mechanism, have been introduced into medical image segmentation. As shown in Figure 3b, GAN consists of a generator and a discriminator. In medical image segmentation, the generator acts as the segmentation network, learning the mapping from medical images to accurate segmentation results; the discriminator evaluates the authenticity of the segmentation results and provides additional supervisory signals to the generator, encouraging it to produce more accurate segmentations. This adversarial training mechanism enables GAN to effectively capture complex features in medical images, improving boundary segmentation quality. Furthermore, GAN can mitigate data imbalance issues by generating more minority-class samples or adjusting the distribution of segmentation results, thereby enhancing the segmentation performance for minority classes.

Conditional GAN (cGAN) [63] was the first to introduce additional conditional information, such as image category labels, patient clinical information, or other related modalities, allowing the generation of samples with specific attributes and features. The architecture consists of a generator and a discriminator. The generator takes both conditional information and random noise as inputs, generating samples that are conditioned on the provided information. The discriminator, on the other hand, not only distinguishes between real and fake samples but also assesses whether the generated sample aligns with the given condition. This conditional constraint mechanism enables cGAN to generate samples with specific attributes and characteristics, significantly enhancing the controllability and relevance of the generated outputs. This, in turn, improves the model’s adaptability and generalization across different medical scenarios.

Compared to traditional methods, cGAN produced segmentation results more closely aligned with the true liver morphology in liver CT image segmentation experiments. Building on this framework, several novel architectures have been proposed. For example, Li et al. [69] introduced a deep learning framework called DiagNet, which uses adversarial sample augmentation to learn highly discriminative features, addressing image classification problems in breast cancer diagnosis. Similarly, Nie et al. [70] proposed adversarial confidence learning for medical image segmentation and synthesis, considering structural information to handle difficult regions, thereby improving the model’s performance in dealing with irregular medical data distributions.

Methods based on cGAN often struggle to capture subtle boundaries due to the use of a single-scale discriminator, resulting in suboptimal performance when segmenting fine structures. To address this issue, Xue et al. [71] proposed a multi-scale discriminator architecture in SegAN. The core of SegAN consists of a generator and a discriminator. The generator employs an encoder–decoder structure to generate segmentation masks, while the discriminator differentiates between the generated segmentation results and the true labels by incorporating multi-scale inputs. This approach enhances the model’s ability to distinguish both global and local structural features. In retinal vessel image segmentation experiments, SegAN demonstrated the ability to clearly segment vascular branches, with an outstanding recall rate and a significant improvement over cGAN.

However, the generator in SegAN lacks sufficient global context modeling, leading to incomplete segmentation in complex background scenarios. To overcome this limitation, He et al. [72] introduced residual connections into the SegAN generator, which alleviates the gradient vanishing problem in deep networks by reusing cross-layer features, thereby enhancing the model’s ability to process global information. He et al. evaluated residual networks with up to 152 layers on the ImageNet dataset, achieving a 3.57% error rate and ranking first in the ImageNet test set, demonstrating the effectiveness of this approach.

Another typical GAN variant, pix2pix [73], introduces the embedding of the entire image as the input to the generator, allowing paired image-to-image translation. The core of pix2pix is conditional GAN, which not only learns the mapping between the input and output images but also learns the loss function required to train this mapping. This allows the same general approach to be applied to solve problems that traditionally require very different types of loss functions. Building on this idea, Ning et al. [74] proposed Pancreas-GAN, a recursive adversarial learning framework for automatic pancreas segmentation, which enforces spatial smoothness consistency across successive image slices by incorporating global distribution constraints. Similarly, Laugh et al. [75] introduced ScarGAN, which uses a chained GAN to simulate scar tissue in healthy myocardium, thereby enhancing segmentation performance. Xing et al. [76] proposed a semi-pixel cyclic GAN (SPCGAN) for robust breast cancer lesion segmentation in 2D ultrasound images. Huo et al. [77] introduced an end-to-end synthetic segmentation network (SynSeg-Net), which trains a segmentation network using unpaired source and target modality intensity images along with manually labeled data from the source modality. This approach achieved good performance in abdominal image synthesis segmentation from MRI to CT for spleen enlargement without requiring manual labels from the target modality.

Supervised learning methods based on GANs, through adversarial training, have significantly improved the authenticity and boundary accuracy of segmentation results, gradually overcoming the limitations of traditional models in segmenting complex structures. Future research could combine GANs with transfer learning to fine-tune pre-trained models using a small amount of labeled target domain data, effectively reducing annotation costs while improving model generalization. Additionally, integrating GANs with reinforcement learning could enable the model to continuously optimize its strategy based on feedback from segmentation results, dynamically adjusting the segmentation process to handle the complex and varying tissue structures and pathological features in medical images. This combination could drive the transition of medical image segmentation technologies from laboratory research to broader clinical applications, ultimately improving patient diagnosis and treatment outcomes.

### 2.5. Other Innovative Methods

Zhang et al. [78] proposed an end-to-end framework that combines deformation modeling and segmentation tasks to achieve single-pass neural anatomical segmentation. Experimental results show that this method significantly outperforms both single-modal and traditional multi-modal segmentation approaches and demonstrates strong robustness on cross-center collected datasets. However, the method has currently only been validated on T1-weighted MRI, and future research should extend its application to other types of MRI scans, such as brain diffusion tensor MRI and infant brain segmentation.

Wang et al. [79] introduced a multi-mixed supervision signal learning (MSL) strategy, MixSegNet, which integrates multiple mixed supervision signals with various network views for training with diverse annotated data. This approach promotes efficient medical image segmentation in real clinical settings. MixSegNet demonstrated outstanding performance on a public MRI cardiac segmentation benchmark dataset, surpassing 21 existing supervised methods, making it a novel and efficient mixed-supervision method for medical image segmentation.

Zhu et al. [80] proposed a triple-knowledge-distillation framework, TED, to enhance knowledge diversity, accuracy, and stability in continuous medical image segmentation tasks, thereby alleviating the problem of catastrophic forgetting. The framework consists of three components: Stochastic Knowledge Augmentation (SKA), Adaptive Knowledge Transfer (AKT), and Global Uncertainty-Guided Fusion (GUGF). In the MRI heart segmentation task, TED achieved an AVG score of 0.896 and a BWT score of 0.031, effectively addressing knowledge transfer issues in multi-task learning. However, the method only considers global uncertainty within the dataset, neglecting the local uncertainty of individual samples. This may result in incorrect weight assignments in certain cases. Future research could explore how to combine both global and local uncertainty to further improve the model’s stability and accuracy.

Test-Time Augmentation (TTA) has emerged as a crucial technique for enhancing the robustness and generalization of medical image segmentation models during deployment. By applying a variety of transformations, such as rotations, flips, and intensity adjustments, to test images during inference, TTA generates multiple augmented versions of the same image. This approach is particularly advantageous in medical imaging, where domain shifts—stemming from variations in imaging protocols, scanners, or patient conditions—can significantly degrade model performance. TTA mitigates these challenges by leveraging the inherent variability of test-time data, thereby improving segmentation accuracy without the need for retraining.

Recent studies have demonstrated the efficacy of TTA across various applications. For instance, Jha et al. [81] integrated TTA with Conditional Random Fields (CRFs), leading to a substantial improvement in segmentation performance using ResUNet++ on colonoscopy images, with enhanced generalization across diverse datasets. Similarly, Karani et al. [82] proposed a test-time adaptable neural network that applies per-image normalization during inference, effectively addressing domain shifts in multi-center prostate MRI data. Furthermore, TTA has been successfully incorporated into U-Net and Mask R-CNN models for cell segmentation in microscopy images, further validating its utility in handling test-time variations [83].

Supervised learning-based medical image segmentation methods, which rely on large amounts of labeled data, can precisely identify different regions within images and provide high-accuracy segmentation results. With the development of deep learning technologies, these methods have continuously evolved. CNNs extract local features through convolution and pooling, but they struggle to capture global information. U-Net enhances the accuracy of segmentation by effectively integrating multi-scale features through its encoder–decoder structure and skip connections. The self-attention mechanism of Transformer addresses the limitations of CNNs when handling large-sized images. GANs generate realistic images through adversarial training, augmenting datasets and improving model generalization capabilities. An increasing number of innovative models combine attention mechanisms and residual structures, further improving the accuracy of segmentation. To assist researchers and clinicians in selecting appropriate methods, Table 4 presents a comparative analysis of 18 representative approaches, covering their key characteristics, advantages, limitations, and applicable scenarios.

## 3. Semi-Supervised Medical Image Segmentation Methods

With the development of deep learning technologies, supervised learning-based segmentation algorithms have made significant progress in medical image segmentation. However, since medical image annotation relies on the knowledge and expertise of specialized doctors, the annotation process is both time-consuming and costly, resulting in a limited amount of labeled data available for training deep learning models [84]. Data scarcity and high annotation costs have become major bottlenecks restricting the widespread application of supervised learning methods in the field of medical image segmentation. As a result, semi-supervised learning has gained considerable attention in medical image segmentation, leading to a large body of research. This section will introduce typical semi-supervised algorithms in the field of medical image segmentation.

### 3.1. Pseudo-Labeling-Based Methods

The pseudo-labeling method is one of the simplest yet most effective approaches in semi-supervised learning [85]. The core idea is to use the model’s predictions on unlabeled data as pseudo-labels, treating them as if they were true labels for training. By iteratively generating pseudo-labels and training the model, this method can progressively leverage the information from unlabeled data to improve segmentation performance. However, if the pseudo-labels contain a significant number of errors, the model may incorporate incorrect information during training, thereby degrading its segmentation performance. Additionally, during the training process, it is crucial to strike a balance between the labeled data and pseudo-label data, ensuring that the model can learn accurate knowledge from labeled data while extracting more information from pseudo-labeled data. Therefore, the key to the pseudo-labeling method lies in generating high-quality pseudo-labels and effectively utilizing them for model training.

To address the issue of insufficient confidence in pseudo-labels in semi-supervised learning, early methods [86] introduced a confidence threshold strategy, where the model outputs a confidence score for each prediction. Only those pseudo-labels with a confidence score above a pre-set threshold are retained and added to the training dataset, thereby filtering out low-confidence predictions. However, the static nature of the threshold setting may result in the exclusion of valuable information. To overcome this limitation, the literature [85] proposed a dual-model ensemble strategy, employing two different network architectures, U-Net and DeepLabV3+, as the base networks for collaborative training, thereby mitigating the bias in confidence estimation from a single model. However, static ensemble methods may not adapt well to dynamic data variations. In response, Shen et al. [87] suggested that the Mean Teacher model can also be viewed as an ensemble pseudo-labeling method, where the predictions from the teacher network serve as more stable pseudo-labels to guide the training of the student network.

Kervadec et al. [88] approached the problem from a different perspective by introducing curriculum semi-supervised learning, which enhances the confidence of pseudo-labels through the incorporation of additional constraints. Experimental results on the ACDC dataset demonstrated that, with only five labeled data samples, this method achieved a Dice coefficient approximately 25% higher than that of fully supervised learning methods, showcasing its strong performance. Similarly, Wu et al. [89] proposed a prototype-based pseudo-label generation method for federated semi-supervised medical image segmentation, where image-level prototypes from labeled data guide the pseudo-label generation on unlabeled client data. This approach effectively addresses the pseudo-label bias caused by cross-center data distribution discrepancies.

To further enhance the reliability of pseudo-labels, Shen et al. [90] designed the Cross-Confidence Supervision Network (CCSM), which reduces the propagation of noisy labels through dual-branch confidence filtering and cross-validation. However, CCSM relies heavily on the quality of the initial pseudo-labels. In response, some studies have proposed self-correcting pseudo-label methods, which iteratively refine the quality of pseudo-labels and use the improved labels to retrain the model. Miao et al. [91] addressed the issue of learning target quality for unlabeled data by proposing a novel self-correcting collaborative training scheme (SC-SSL). This method enables the model to learn targets that are closer to true labels, thereby more effectively exploring unlabeled data with semantic context awareness.

In the field of medical image segmentation, noise in pseudo-labels can lead to a decline in model performance, while constrained pseudo-labels often suffer from insufficient information. To address these challenges, Min et al. [92] introduced the Deep Attention Network (DAN), which can adaptively detect and correct errors in noisy labels, thereby enhancing the quality of pseudo-labels. However, DAN is sensitive to data transformation methods, and training two student networks simultaneously increases computational load and training time, impacting efficiency. To overcome these limitations, future research could explore more stable data transformation strategies to reduce dependence on specific transformations and optimize network architectures through techniques such as model pruning and quantization, thereby improving training efficiency.

The application of pseudo-labeling in semi-supervised learning continues to evolve in medical image segmentation, with advancements ranging from early confidence threshold strategies to dual-model ensembles, curriculum-based semi-supervised learning, and more complex models like self-correcting pseudo-labels and deep attention networks. Each innovation has contributed to improving pseudo-label noise and information deficiency. Despite these advances, challenges remain, including reliance on initial label quality, sensitivity to data transformations, and high computational cost. Future research should focus on overcoming these bottlenecks to enhance the efficiency and accuracy of pseudo-labeling methods in medical image segmentation.

### 3.2. Consistency Regularization-Based Methods

The core idea of consistency regularization is based on the assumption that a model should produce consistent predictions for unlabeled data after undergoing different perturbations [93]. This approach encourages the model to learn robust feature representations that are insensitive to input variations, thereby leveraging the information from unlabeled data to enhance the model’s generalization capability. Consistency regularization has garnered widespread attention in the field of medical image segmentation, leading to various implementations of this concept.

Early methods of consistency regularization faced limitations in fully exploiting the potential of unlabeled data. To address this, data perturbation-based consistency regularization methods were introduced, which apply various perturbations to the input data, such as data augmentation and noise injection, to force the model to generate consistent segmentation results across different data views [94]. This approach enables a deeper exploration of the information contained in unlabeled data. Building on this idea, You et al. [95] proposed the ARCO (Adaptive Rectified Contrastive Learning) framework to address the common issues of long-tail distribution and class imbalance in medical image data. Experimental results demonstrated that ARCO outperformed previous semi-supervised methods on several medical image segmentation datasets. Similarly, Bai et al. [94] introduced the Bidirectional Copy–Paste (BCP) method, where unlabeled data learn comprehensive semantic information from labeled data, and labeled data benefit from the knowledge extracted from unlabeled data, effectively reducing the distribution gap between the two. However, the effectiveness of this method may be limited in more complex scenarios and tasks, suggesting that future work could explore more sophisticated regularization functions or incorporate additional prior knowledge to further enhance its performance.

Consistency regularization based on data perturbation modifies the inherent features of the original data, which, in medical imaging, may distort true anatomical structures and impact the accuracy of segmentation. In contrast, model perturbation-based consistency regularization overcomes this limitation by focusing on the structure and parameters of the model itself. It applies various perturbation techniques, such as dropout [96] and stochastic depth [97], to perturb the model, generating different model views. The model is then required to produce consistent predictions for the same input across these different perturbed views.

**Figure 6:**
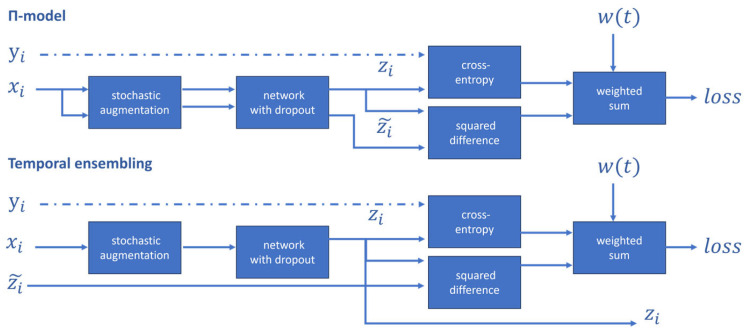
The architecture of Π-model and Temporal Ensembling [98].

Laine et al. [98] proposed the Π-model and Temporal Ensembling frameworks, based on the idea of model perturbation for consistency regularization, as illustrated in Figure 6. The Π-model applies the same or different dropout perturbations twice to the same input, requiring the model to produce consistent predictions for the same unlabeled data sample after undergoing different transformations. Temporal Ensembling, on the other hand, uses the exponentially moving average (EMA) of historical predictions as a consistency target to constrain the current prediction, encouraging the model to learn feature representations that are invariant to data transformations, thereby enhancing its generalization capability. During training, the model simultaneously employs supervised learning with labeled data and consistency regularization learning with unlabeled data. By minimizing the supervised loss on labeled data and the consistency loss on unlabeled data, the model can effectively leverage the information from unlabeled data even when labeled data are scarce, improving their generalization performance.

**Figure 7:**
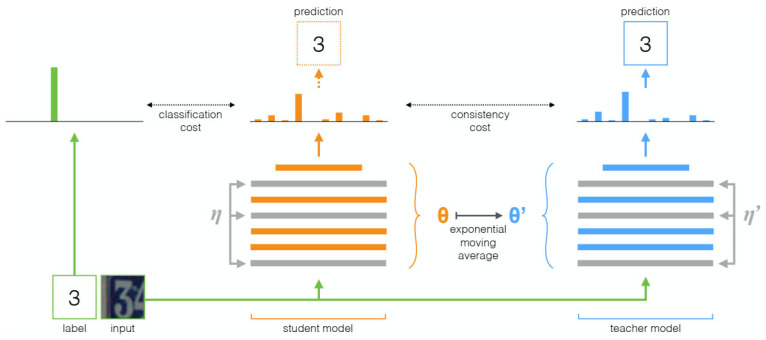
The Mean Teacher Method [99]. During computational processes, both the student model and the teacher model assess the input by incorporating noise. The SoftMax output generated by the student model is contrasted with the one-hot label through the application of classification cost, and it is also compared with the output of the teacher model using consistency cost. Once the weights of the student model have been adjusted via gradient descent, the weights of the teacher model are then updated in the form of an exponential moving average of the student model’s weights.

Inspired by the Π-model and Temporal Ensembling, Tarvainen et al. [99] introduced the Mean Teacher method, which combines the concepts from both approaches. Compared to traditional target network training methods, this approach computes the running average of the student model’s weights as the teacher model’s weights during training, using the teacher model’s predictions as the reference target for the student. This improves the accuracy and robustness of the target network. Additionally, the Mean Teacher method introduces random noise to both the input and output of the student model during each training iteration, enhancing the model’s generalization ability. The target network is optimized by minimizing the distance between the predictions of the student model and those of the teacher model. The training framework is illustrated in Figure 7. When applied to the SVHN dataset with 250 labels, the method achieved an error rate of only 4.35%, outperforming Temporal Ensembling, which used 1000 labels, demonstrating its efficient use of unlabeled data.

In the Mean Teacher framework, the teacher model’s generated targets may contain noise and unreliability due to the lack of labeled data, leading the student model to learn incorrect information, which negatively impacts the effectiveness of semi-supervised learning. To address this issue, Yu et al. [100] proposed the uncertainty-aware semi-supervised learning framework, UA-MT, based on Mean Teacher. This approach not only improves the accuracy of segmentation but also reduces the need for labeled data, thus saving on labor costs. Ouali et al. [101] combined the EMA strategy from Mean Teacher with a method called Cross-Consistency Training (CCT). Experimental results on the PASCAL VOC dataset showed that CCT outperformed traditional methods with different numbers of labeled samples, achieving up to a 21-point improvement in mIoU.

However, the method’s performance is compromised in low-density regions. Sohn et al. [102] developed a simple and efficient consistency regularization framework, FixMatch, by incorporating differentiated augmentation perturbations and high-confidence pseudo-label filtering. This method maintains prediction consistency under various perturbations, focusing on high-confidence regions, and reduces the risk of error label propagation, making it particularly suitable for medical imaging scenarios with blurred boundaries or high noise levels.

To further optimize the effectiveness of consistency regularization, some research has started focusing on adaptive consistency learning, which dynamically adjusts the strength or approach of consistency regularization based on the characteristics of the data or the model’s state [103]. This allows for more flexible utilization of unlabeled data. Based on this idea, Wu et al. [103] proposed SS-Net, which simultaneously explores pixel-level smoothness and inter-class separation to address issues such as the scarcity of labeled data and blurred boundaries in medical image segmentation. However, when the dataset suffers from class imbalance, the model tends to overly focus on the majority class and neglect the minority class, which negatively impacts overall classification accuracy. To address the limitations of SS-Net, You et al. [104] introduced the ACTION++ framework, which incorporates adaptive supervised contrastive loss to encourage features from different classes to match different, evenly distributed class centers. Experimental results demonstrate that this method outperforms others on the ACDC and LA benchmarks, proving its effectiveness and reliability in practical medical image segmentation tasks, and highlighting its high application value.

In the field of medical image segmentation, semi-supervised methods based on consistency regularization help the model extract stable features that are truly relevant to the disease by perturbing either the data or the model itself. This process ensures that the model focuses on features that are not influenced by noise or individual variations in the images, thereby improving diagnostic accuracy and reliability. These methods have demonstrated great potential both in theoretical research and practical applications. Different approaches have achieved unique results in addressing their respective challenges.

Table 5 provides a detailed quantitative comparison of key metrics, such as the Dice coefficient, Jaccard index, 95% Hausdorff Distance (HD95), and Average Surface Distance (ASD), for heart segmentation tasks, offering a clear performance comparison of these methods in practical applications. BCP achieved the highest Dice score (89.62%) and Jaccard index (81.31%), along with the lowest HD95 (6.81) and ASD (1.76), indicating the superior accuracy of segmentation. Other methods like SS-Net and UA-MT also performed well, with Dice scores exceeding 87%, demonstrating their effectiveness in extracting robust features from both labeled and unlabeled data.

Notably, methods incorporating a higher percentage of labeled data, such as URPC (10% labeled) and MC-Net, showed improved segmentation performance compared to those relying on fewer labeled samples (URPC with 5%), suggesting that a moderate amount of labeled supervision still plays a crucial role in optimizing semi-supervised models. These findings reinforce the importance of balancing labeled and unlabeled data while integrating advanced regularization techniques to enhance model robustness and generalization in medical image segmentation.

Semi-supervised methods based on consistency regularization complement each other in terms of technical implementation and data processing, collectively forming a technical framework for consistency regularization. However, they also face numerous challenges, such as sensitivity to hyperparameters, significant variations in medical image data, and high computational resource requirements. Future research could focus on developing adaptive hyperparameter adjustment algorithms, employing techniques like data augmentation and domain adaptation to enable the model to learn invariant features across different data distributions. Additionally, designing lightweight model architectures could help improve training and inference speed while maintaining performance, providing new ideas and technical support for the advancement of medical image segmentation.

In this context, pseudo-labeling and consistency regularization exhibit distinct strengths in low-resource settings. Pseudo-labeling (e.g., DAN [92]) leverages high-confidence predictions to expand labeled data, but it is vulnerable to annotation noise. For example, in the WORD dataset, a 30% synthetic noise level caused a 12% drop in the Dice score. In contrast, consistency regularization methods (e.g., FixMatch [102]) enforce prediction invariance under perturbations, showing only a 5% performance decline under the same noise level. This highlights the robustness of consistency regularization in noisy environments. However, pseudo-labeling excels in extreme low-label scenarios, such as when only 5% of the data is labeled. In such cases, pseudo-labeling achieved a Dice score of 78.4% on the LA dataset, likely due to its direct utilization of unlabeled data confidence. These findings suggest that a hybrid approach combining both methods could optimally balance robustness and efficiency, making it a promising direction for future research.

### 3.3. Generative Model-Based Methods

Generative models, particularly Generative Adversarial Networks (GANs), play a unique role in semi-supervised learning. GANs can generate more realistic medical image data to expand training datasets, alleviating the problem of data scarcity. They can also function as discriminators to assist in training segmentation models, improving the quality and realism of segmentation results through adversarial learning.

Data augmentation is a key strategy in semi-supervised medical image segmentation based on generative models. By using GANs to generate realistic synthetic medical images and combining these synthetic images with real labeled data, training datasets can be expanded, thus enhancing the performance of segmentation models. Based on this idea, Mondal et al. [105] used GANs to generate synthetic medical image data for segmenting 3D multi-modal medical images with very limited labeled data, demonstrating good performance on a brain MRI dataset. Madani et al. [106] employed GANs to generate chest X-ray images to augment the dataset for training disease classification models, achieving a significant improvement in the recognition accuracy of various lung diseases, offering a new solution for reducing the cost of medical data acquisition. Kugelman et al. [107] used data augmentation with GANs and semi-supervised learning to enhance retinal and choroidal layer segmentation in Optical Coherence Tomography (OCT) images, improving classification performance and boundary delineation, which has significant clinical implications.

To address the issue of early convergence of a single discriminator, which makes it difficult for the generator to continue learning and improving generation quality, a dual-discriminator structure introduces a second discriminator focused on global feature discrimination, significantly enhancing the effectiveness of adversarial learning. Zhu et al. [108] proposed a cyclic consistency architecture, CycleGAN, built on two generators and two discriminators, which can generate medical image data with different characteristics, thereby improving the generalization ability of models in medical image segmentation tasks.

Based on this architecture, Xu et al. [109] introduced the Semi-Supervised Attention-Guided CycleGAN (SSA-CycleGAN), which is used to synthesize tumor images from normal images and reconstruct normal images from tumor images. Similarly, Qi et al. [110] proposed a novel CycleGAN-based data augmentation model, SAG-GAN, which uses a semi-supervised attention mechanism to guide the generation of abnormal images, addressing the issue of data scarcity in brain tumor classification tasks. Zhang et al. [111] proposed a semi-supervised medical image segmentation method based on adversarial consistency learning and dynamic convolution. By employing a dual-discriminator structure, this method more comprehensively leverages the advantages of adversarial learning, enhancing semi-supervised segmentation performance. Zhou et al. [112] introduced a weakly supervised medical image segmentation model based on deep generative models, which reduces the dependence on pixel-level labels for lesion segmentation in medical images, assisting healthcare professionals in achieving faster diagnoses.

Semi-supervised learning aims to train models using a small amount of labeled data and a large amount of unlabeled data, thereby reducing labeling costs while improving model performance and generalization ability. The rapid development of deep learning techniques has injected new vitality into semi-supervised medical image segmentation [113,114]. Different semi-supervised methods vary in how they utilize data, generate pseudo-labels, and depend on labeled data. Table 6 provides a comprehensive comparison of 14 semi-supervised methods, offering insights into their characteristics and applications.

## 4. Unsupervised Medical Image Segmentation Methods

In the field of medical image segmentation, the high cost and expertise required for data annotation have long limited the practical application of supervised methods. Semi-supervised learning, which integrates a small amount of labeled data with a large amount of unlabeled data, has become an important approach to alleviate the annotation bottleneck. However, semi-supervised methods rely on an initial “seed” of labeled data, making them susceptible to the quality of annotations and domain shift. In cases of extreme annotation scarcity, their performance is further constrained. As a result, researchers have begun to explore unsupervised learning. Unsupervised learning does not depend on manual annotations but instead automatically learns useful representations and patterns from the data itself, enabling knowledge acquisition and transfer. This section will introduce typical unsupervised algorithms in the domain of medical image segmentation.

### 4.1. Unsupervised Domain Adaptation Methods

In the field of medical image segmentation, unsupervised learning has primarily focused on the task of Unsupervised Domain Adaptation (UDA). The core idea of UDA is to leverage labeled information from the source domain and unlabeled information from the target domain to learn domain-invariant feature representations, thereby reducing the discrepancy between the source and target domains. This allows models to transfer from the source domain, which has abundant labeled data, to the target domain, which lacks labeled data, while maintaining or closely approximating the performance achieved in the source domain. In recent years, deep learning-based unsupervised domain adaptation methods have made significant progress in medical image segmentation tasks. This section will introduce image alignment-based unsupervised domain adaptation methods, Fourier transform-based image style transfer methods, and unified unsupervised domain adaptation frameworks.

#### 4.1.1. Image Alignment-Based Unsupervised Domain Adaptation Methods

Image alignment-based unsupervised domain adaptation methods aim to reduce the appearance gap between the source and target domains by using image transformation techniques to transfer the style of source domain images to the target domain, or vice versa. The goal of image alignment is to enable the segmentation model to learn domain-independent semantic information. Generative Adversarial Networks (GANs), as a powerful generative model, have achieved great success in image style transfer tasks, making them widely used in image alignment-based unsupervised domain adaptation methods.

To effectively reduce the domain gap between the source and target domains, several studies have proposed bidirectional image style transfer strategies based on GANs. Chen et al. [115] introduced the Synergistic Image and Feature Alignment (SIFA) framework, which performs domain alignment at both the image and feature levels. In cardiac substructure segmentation tasks, the SIFA method achieved an average Dice score greater than 70% across four cardiac structures, demonstrating a significant performance improvement and proving to be an effective unsupervised domain adaptation approach.

Similarly, Han et al. [116] proposed the Deep Symmetric Adaptation Network (DSAN) for cross-modal medical image segmentation. The method consists of two main components: a feature alignment subnetwork and a semantic mining subnetwork, which enable bidirectional feature alignment and the extraction of additional semantic information. Experimental results show that DSAN achieved excellent performance across multiple cross-modal medical image segmentation tasks, including cardiac, brain tumor, and abdominal multi-organ segmentation, with Dice coefficients improving by nearly 10%. Zou et al. [117] proposed the Dual-Scheme Fusion Network (DSFN), which performs collaborative alignment of the source and target domains from both the image and feature levels. Compared to other advanced domain adaptation methods, DSFN demonstrated significant performance improvements.

Bidirectional image style transfer methods based on GANs, by learning image translation models from both the source domain to the target domain and vice versa, enable a more comprehensive capture of domain differences and allow for more effective domain alignment through bidirectional migration. However, the image style transfer process itself may introduce noise or alter the semantic information of the original image, potentially affecting the performance of segmentation models. Ensuring semantic consistency during image style transfer is a critical concern for domain adaptation methods based on image alignment.

Several studies have addressed this by incorporating various constraints or prior knowledge, such as semantic consistency constraints, anatomical structure priors, and frequency domain constraints, to improve the quality and semantic coherence of image translation. Luo et al. [118] proposed the Self-Improved Domain Adaptation (SIDA) method, which enhances the effectiveness and robustness of the image translation module for segmentation networks by employing two self-supervised tasks. In unsupervised domain adaptation for pancreatic segmentation (CT-MRI), the SIDA method achieved a Dice coefficient improvement of approximately 6% over SIFA, demonstrating its effectiveness and superior performance. Zhuang et al. [119] introduced an Anatomy-Guided Self-Training Segmentation Framework (ASTCMSeg) for unpaired cross-modal medical image segmentation. By incorporating anatomical structure prior knowledge, ASTCMSeg generates more semantically consistent and structurally coherent translated images, thereby improving the performance of subsequent segmentation models. ASTCMSeg demonstrated advanced performance in cross-modal brain structure, cardiac substructure, and abdominal multi-organ segmentation tasks.

#### 4.1.2. Fourier Transform-Based Image Style Transfer Methods

Unsupervised domain adaptation methods based on image alignment leverage image transformation techniques to reduce the appearance gap between the source and target domains by transferring image styles between them. This allows the segmentation model to learn domain-invariant semantic information, thereby enhancing the generalization performance on the target domain, achieving remarkable results in medical image segmentation. However, traditional methods may face limitations when dealing with complex textures and high-frequency information. Fourier transform, which converts an image from the spatial domain to the frequency domain, decomposes the image into components of different frequencies. Fourier transform-based image style transfer methods can facilitate style transfer by exchanging or adjusting the frequency components of the source and target domain images, while preserving the structural information of the image as much as possible. This provides an efficient feature alignment solution for medical image segmentation.

In 2020, Yang et al. [120] proposed Fourier Domain Adaptation (FDA), which operates on the data in the Fourier domain to achieve image style transfer by exchanging the low-frequency components of the source and target domain images. This approach introduced new ideas and methods for unsupervised domain adaptation research. Building on this concept, Oh et al. [121] introduced the FIESTA method, which enhances single-source domain generalization in medical image segmentation tasks. Similarly, Xian et al. [122] proposed the Dual Adaptation-Guiding Network (DAG-Net) for 3D medical image segmentation. DAG-Net consists of two modules: the Fourier-based Contrastive Style Augmentation (FCSA) module and the Residual Space Alignment (RSA) module. In cross-modal transfer tasks for cardiac substructure and abdominal multi-organ segmentation, DAG-Net outperformed existing domain adaptation methods.

#### 4.1.3. Unified Unsupervised Domain Adaptation Framework

MAPSeg [123] is a highly versatile and performant unified UDA framework designed to address heterogeneity and volumetric issues in medical image segmentation. The method consists of three components: (1) a 3D multi-scale masked autoencoder (MAE) for self-supervised pretraining—by randomly masking parts of the image, the model is disturbed in a way that enhances its generalization ability, enabling it to adapt to different scenarios and tasks; (2) a 3D masked pseudo-label (MPL) for domain-adaptive self-training—pseudo-labels are generated based on the predictions for target domain data and incorporated into the training set, allowing the model to better align with the target domain’s data distribution; and (3) Global–Local Feature Fusion (GLC)—this module leverages both global and local context relationships, connecting local and global semantic features in latent space and making predictions based on the fused features, further enhancing segmentation performance. Experimental results across multiple domain shift and adaptation scenarios demonstrate that MAPSeg can effectively handle cross-sequence, cross-site, cross-age, and cross-modal domain shift issues in medical image segmentation. It can be applied in centralized, federated, and test-time UDA settings, offering significant practical value in the medical image segmentation field.

### 4.2. Contrastive Learning-Based Unsupervised Segmentation Methods

In the field of medical image segmentation, unsupervised domain adaptation methods aim to address the challenge of inconsistent data distributions between the source and target domains. By employing strategies such as image alignment and adversarial training, these methods transfer knowledge from the source domain model to the target domain, thereby improving segmentation performance in the target domain [124]. While notable progress has been made, traditional unsupervised domain adaptation methods face limitations in extracting intrinsic semantic information and in learning discriminative feature representations. In response, contrastive learning-based unsupervised segmentation methods have emerged, emphasizing the learning of more discriminative feature representations by contrasting the similarities and differences between different samples. This approach offers a new perspective and powerful techniques for medical image segmentation, leading to breakthroughs in tackling the complex and dynamic challenges of medical image segmentation.

Jiang et al. [125] proposed Prototypical Contrastive Adaptation (ProCA), which combines contrastive learning with domain adaptation. By optimizing feature representations using contrastive losses between positive and negative sample pairs, ProCA reduces the distributional gap between the source and target domains. Gao et al. [126] introduced an unsupervised representation learning framework for pathological tissue image segmentation, addressing the challenge of limited tissue segmentation samples by designing three contrastive learning tasks at the image, superpixel, and pixel levels. Liu et al. [127] developed CLMorph, an unsupervised medical image segmentation method based on contrastive registration. This method achieves high-accuracy segmentation through image-level registration and feature-level contrastive learning, making it suitable for multimodal medical image analysis. It is robust and adaptable, although it relies on registration accuracy when dealing with complex anatomical structures.

The above contrastive learning-based unsupervised segmentation methods focus on optimizing feature learning and segmentation performance by emphasizing image-specific features or inter-image contrast relationships, through approaches such as category prototype contrast, multi-granularity view contrast, and contrastive registration. However, the medical field contains rich textual information, such as medical reports and clinical records, which, if effectively utilized, could enhance medical visual representations. MLIP [128] is a novel medical visual representation enhancement framework that uses textual information from medical reports as auxiliary signals for unsupervised pretraining, strengthening the model’s understanding and reasoning capabilities. This enables more accurate and effective medical image-text contrastive learning, achieving excellent performance in enhancing transferability and surpassing existing methods even with limited annotated data. MLIP offers new insights for medical representation learning.

### 4.3. SAM-Based Segmentation Methods

The Segment Anything Model (SAM) [129], introduced by Meta AI Research in 2023, is a powerful image segmentation foundation model capable of adapting to new tasks and distributions without requiring any labeled data. SAM was trained on a large amount of high-quality auto-labeled data, granting it strong zero-shot segmentation capabilities. This provides a novel solution to the challenge of scarce manually labeled images in medical image segmentation, garnering widespread attention from researchers in the field.

Ma et al. [130] were the first to apply SAM to medical image segmentation, creating a universal model called MedSAM. This model combines SAM’s robust generalization ability with domain-specific medical knowledge, offering higher versatility and flexibility, thus improving segmentation performance. Cheng et al. [131] conducted a comprehensive fine-tuning of SAM, introducing SAM-Med2D for 2D medical image segmentation tasks, and demonstrated its strong generalization capabilities across multiple datasets. However, SAM-Med2D struggles to capture the spatial information inherent in 3D medical images. To address this, Wang et al. [132] extended SAM’s encoder and decoder to 3D, proposing SAM-Med3D, which supports the extraction of 3D spatial features from modalities such as CT and MRI. Experimental results on 15 datasets showed that SAM-Med3D improved the Dice coefficient by an average of 12% in 3D segmentation tasks, significantly enhancing SAM’s ability to capture 3D spatial information.

SAM-based methods have demonstrated significant potential in medical image segmentation by unlocking the value of data and enhancing segmentation performance. This highly automated and accurate segmentation approach represents a future research trend in the field of medical image segmentation. Further exploration of SAM is expected to elevate the field, advancing clinical diagnosis and treatment.

Unsupervised methods in medical image segmentation aim to extract the intrinsic structure and features of the data itself to achieve segmentation without the need for labeled data, thus eliminating the labor-intensive annotation process. However, there remains a gap in the accuracy of segmentation and stability compared to supervised methods. Table 7 summarizes 14 representative methods, highlighting their core features, advantages, limitations, and applicable scenarios, providing valuable insights for research and application in the field.

## 5. Commonly Used Datasets, Evaluation Metrics, and Loss Functions

### 5.1. Common Medical Image Datasets

The performance validation and generalization capability of medical image segmentation algorithms heavily rely on high-quality, diverse labeled datasets. However, the acquisition of medical image data faces three core challenges: multi-modal heterogeneity, anatomical complexity, and the professional barriers to annotation. To address these issues, researchers have collaborated across institutions to construct standardized segmentation datasets that cover major anatomical regions and imaging modalities, providing a repeatable benchmarking platform for algorithm development. As shown in Table 8, this paper systematically organizes mainstream datasets that include key anatomical areas such as the abdomen, chest, brain, eye, kidney and pancreas, covering major imaging techniques like CT, MRI, and endoscopy. The annotation scope ranges from single-organ to multi-organ segmentation, offering multidimensional benchmark support for algorithm development.

### 5.2. Evaluation Metrics

In the field of medical image segmentation, accurately evaluating model performance and appropriately selecting loss functions are critical for model training and optimization. Commonly used evaluation metrics for assessing the effectiveness of segmentation algorithms are as follows:

Recall: This metric represents the proportion of true positive samples among all actual positive samples. In the context of detecting lesions in medical images, a high recall rate indicates the model’s ability to identify as many of the actual lesion pixels as possible. Therefore, this metric is particularly important in lesion detection tasks.

(1) $$Recall=\frac{TP}{TP+FN}.$$

Dice Coefficient: The Dice coefficient measures the degree of overlap between two regions, with values ranging from [0, 1], where a value closer to 1 indicates a higher overlap between the segmentation result and the ground truth. The Dice coefficient is robust to class imbalance and is one of the most commonly used evaluation metrics in medical image segmentation. It is widely applied in the segmentation evaluation of various organs and lesions.

(2) $$Dice=\frac{2TP}{2TP+FP+FN}\;.$$

Intersection over Union (IoU): IoU is the ratio of the intersection to the union of the predicted and ground truth regions, with values ranging from [0, 1]. IoU intuitively reflects the degree of overlap between the segmentation result and the ground truth. It is commonly used for evaluation in object detection and image segmentation tasks. In medical imaging, IoU can be used to assess the quality of organ or lesion segmentation.

(3) $$IoU=\frac{TP}{TP+FP+FN}\;.$$

Hausdorff Distance (HD): Hausdorff distance is used to measure the maximum degree of mismatch between two point sets. In medical image segmentation, it reflects the maximum distance between the predicted segmentation contour and the ground truth contour.

(4) $$HD\left(A,B\right)=max\left\{h\left(A,B\right),h\left(B,A\right)\right\},$$

(5) $$h\left(A,B\right)=\underset{a\in A}{\max}\underset{b\in B}{\min}\left|\left|a-b\right|\right|.$$

Mean Absolute Error (MAE): MAE calculates the average of the absolute differences between the predicted segmentation result and the ground truth for each pixel. It measures the average deviation between the predicted and true values. Let $x_{i}$ be the predicted value, $y_{i}$ the true value, and n the total number of pixels. A smaller MAE value indicates that the segmentation result is closer to the ground truth.

(6) $$MAE=\frac{1}{n}\sum_{i=1}^{n}\left|x_{i}-y_{i}\right|\;.$$

Accuracy of Segmentation: the proportion of correctly segmented regions among all predictions

### 5.3. Loss Functions

In addition to the network architecture, one of the essential characteristics of deep learning models is the loss function [154]. The commonly used loss functions in the field of medical image segmentation are as follows:

Cross-entropy loss: for binary classification problems, the cross-entropy loss function is:

(7) $$L=-\sum_{i=1}^{N}\left\{y_{i}\log\left(p_{i}\right)+\left(1-y_{i}\right)\log\left(1-p_{i}\right)\right\},$$

Here, $y_{i}$ represents the ground truth label, and $p_{i}$ is the predicted probability. For multi-class problems, there is an extended form of the cross-entropy loss. Cross-entropy loss effectively measures the difference between the predicted probability distribution and the ground truth label distribution, encouraging the model to output probabilities closer to the ground truth labels. It is commonly used in both classification and segmentation tasks.

Dice Loss: derived from the Dice coefficient, with GT (Ground Truth) as the reference value and SR (Segmentation Result) as the segmentation outcome.

(8) $$Dice=\frac{2\left|GT\cap SR\right|}{\left|GT+SR\right|}.$$

Tversky Loss is an improved loss function based on Dice Loss, which assigns different weights to false positives and false negatives. In medical imaging, class imbalance is a common issue, where the target regions (e.g., lesion areas) occupy a small proportion of the entire image. Tversky Loss effectively addresses this imbalance by adjusting the weights, enhancing the model’s ability to segment small targets.

(9) $$L=\frac{\left|GT\cap SR\right|}{\left|GT\cap SR\right|+\alpha \left|SR\backslash GT\right|+\beta \left|GT\backslash SR\right|}\;.$$

## 6. Discussion

### 6.1. Summary of Deep Learning-Based Medical Image Segmentation Methods

#### 6.1.1. Supervised Deep Learning-Based Medical Image Segmentation Methods

Supervised learning, as the mainstream approach in medical image segmentation, demonstrates significant performance advantages in recognizing complex anatomical structures and locating lesions by leveraging strong supervision signals from labeled data through deep neural networks. This summary reveals three key trends in current technological development: architectural innovation driving performance breakthroughs, the trade-off optimization between computational efficiency and accuracy, and improved adaptability to clinical scenarios. Specifically, (1) methods based on attention mechanisms and Transformer architectures enhance global context modeling, achieving sub-pixel accuracy in tasks such as brain imaging and polyp segmentation; (2) to address computational resource constraints, nested skip connections and cyclic residual structures reduce redundant computation through feature reuse strategies, while models like Swin-UNet balance computational efficiency and model capacity via hierarchical window attention; (3) regarding scene adaptability, ICGNet strengthens low-contrast boundary detection through an inverse contour guidance module, while UM-Net mitigates illumination variation interference using color transfer operations, reflecting a customized design approach for specific imaging defects.

#### 6.1.2. Deep Learning-Based Semi-Supervised Medical Image Segmentation Methods

Semi-supervised learning methods, by leveraging both limited labeled data and vast amounts of unlabeled data, exhibit significant advantages in labeling efficiency for medical image segmentation tasks. We have identified two major directions in current technological development: pseudo-label quality optimization and consistency constraint enhancement. In terms of pseudo-label optimization, early algorithms reduced noise interference by applying confidence thresholds, while DAN improved pseudo-label reliability through an adaptive noise correction module. BCP, on the other hand, mitigated long-tail distribution issues using a bidirectional copy–paste strategy. These approaches break through performance bottlenecks from the perspectives of sample selection, noise suppression, and data distribution balancing. Regarding consistency constraints, FixMatch builds a prediction consistency objective by combining strong and weak data augmentation, Mean Teacher stabilizes teacher model outputs through weight averaging, and UA-MT introduces an uncertainty-aware mechanism to dynamically adjust the supervision strength. These methods enhance model generalization by modeling invariance to perturbations in different forms.

#### 6.1.3. Deep Learning-Based Unsupervised Medical Image Segmentation Methods

Unsupervised learning methods, by extracting intrinsic semantic relationships and domain-invariant features from data, provide innovative solutions to the challenges of limited medical image annotations and cross-domain generalization. In recent years, unsupervised learning algorithms have evolved along three main technical directions: domain-adaptive feature alignment, contrastive semantic enhancement, and frequency-domain decoupling and reconstruction. In the domain adaptation aspect, SIFA achieves dual alignment of images and features through a dual-path collaborative optimization, DSAN separates domain-invariant or domain-specific features by utilizing a shared encoder and private decoder, while DAG-Net constructs a cross-modal spectral adaptation module based on Fourier transform. These methods break through the domain shift bottleneck from pixel-level, feature-level, and frequency-domain perspectives.

Meanwhile, SAM’s powerful segmentation capabilities provide new insights for the medical image field. Under the unsupervised learning framework, SAM can be used to more accurately initialize segmentation masks, providing a more reliable foundation for subsequent unsupervised domain adaptation feature alignment tasks. In contrastive learning-driven methods, ProCA enhances class discriminability through prototype contrastive constraints, CLMorph improves cross-modal generalization by combining contrastive learning and image registration, and MLIP integrates medical prior knowledge to guide the construction of contrastive feature spaces, reflecting the emerging trend of “data-driven + knowledge-guided” fusion. Additionally, frequency-domain methods achieve fast domain adaptation with low computational overhead by exchanging low-frequency information or uncertainty-guided frequency-domain enhancement. However, their ability to preserve details and their multi-source generalization still require further optimization.

### 6.2. Challenges in Current Medical Image Segmentation Methods

Despite significant advancements in deep learning-based medical image segmentation, several critical challenges persist, limiting the robustness, efficiency, and clinical applicability of existing methods. These challenges stem from both inherent characteristics of medical imaging data and technical limitations of current algorithms. Below, we systematically analyze these challenges with supporting evidence from recent studies.

#### 6.2.1. Limited Generalization Across Domains

Medical images exhibit substantial variability across different imaging modalities (e.g., MRI, CT, ultrasound), acquisition protocols, and institutions. For instance, cardiac MRI scans from different devices may differ in resolution, contrast, and artifact patterns, resulting in domain shifts that negatively affect model performance. Unsupervised domain adaptation (UDA) techniques seek to mitigate these issues by aligning feature distributions across domains. However, as highlighted in Section 4.1, these methods frequently fail to maintain anatomical consistency during style transfer, which leads to the suboptimal accuracy of segmentation. Additionally, models trained on single-modal datasets (e.g., 2D U-Net) often exhibit limited generalizability when applied to 3D or multi-modal contexts, thus restricting their clinical applicability.

#### 6.2.2. Challenge of Overfitting in Medical Image Segmentation

Overfitting presents a critical challenge in medical image segmentation, where models demonstrate high accuracy on training data but struggle to generalize to unseen data. This issue arises when the model complexity exceeds the diversity and size of the available training dataset, often exacerbated by the scarcity of annotated medical images. In medical imaging, the problem is further amplified by variations in imaging modalities, patient demographics, and disease characteristics.

To mitigate overfitting, regularization techniques such as L2 regularization are commonly applied, which penalize large model weights, thereby reducing the risk of overfitting by preventing the model from memorizing noise or irrelevant features. Dropout, by randomly deactivating a subset of neurons during training, encourages more robust feature learning and reduces reliance on specific pathways. Data augmentation, involving transformations such as rotation, scaling, and intensity adjustments, effectively enlarges the training dataset, improving the model’s ability to generalize across diverse real-world conditions. When combined, these techniques significantly enhance the model’s performance, robustness, and clinical applicability.

#### 6.2.3. The Computational Cost of the Proposed Methods

Training deep learning models, particularly those with large parameter spaces, remains computationally intensive and time-consuming. This challenge is exacerbated when applying these models to large-scale medical datasets or performing inference on high-resolution 3D images. For instance, Transformer-based architectures such as UNETR [62] and Swin-UNet [65], while achieving state-of-the-art segmentation performance in tasks like brain tumor segmentation, require substantial GPU memory and prolonged training times due to their global self-attention mechanisms and multi-scale feature fusion. Similarly, 3D U-Net [55], though effective for volumetric data, incurs significant computational overhead during both training and inference, which limits its applicability in resource-constrained clinical settings.

To improve efficiency, lightweight models such as CoTr [63] and ICGNet [29] have been proposed. CoTr utilizes a sparse attention mechanism to reduce computational complexity by approximately 30%, while maintaining competitive performance (e.g., a 3% improvement in Dice scores on the BCV dataset). ICGNet incorporates lightweight modules, such as the Adaptive Local–Global Context Module (ALGM), to enhance computational efficiency, enabling real-time inference on colonoscopy images with minimal accuracy trade-offs. However, these methods still face a fundamental trade-off: simplifying model architectures often compromises the ability to capture fine-grained anatomical details or generalize across heterogeneous datasets.

### 6.3. Development Trends in Deep Learning-Based Medical Image Segmentation Methods

With the widespread application of deep learning techniques in medical image segmentation, their value in clinical diagnosis and treatment planning has become increasingly prominent. However, challenges such as modality diversity, high annotation costs, complex target structures, and insufficient model generalization remain core issues in current research. As a result, most computer-aided diagnostic models have yet to see practical implementation and widespread adoption in clinical practice. Based on existing technological advancements and clinical needs, we believe that future research directions should focus on the following key trends:

#### 6.3.1. Deepening of Semi-Supervised and Unsupervised Learning

With the continuous advancement of medical imaging technology, the volume of generated data has grown exponentially, presenting significant challenges in acquiring annotated data. Data scarcity remains a long-standing limiting factor in the development of this field. To address this issue, future research will focus on the deep optimization of semi-supervised and unsupervised learning paradigms to reduce the model’s over-reliance on annotated data. In the domain of semi-supervised learning, large models’ robust feature extraction and generalization capabilities can be leveraged, combining a small amount of annotated data with a large amount of unannotated data for model training. For instance, a large model can be pre-trained on a small annotated dataset through supervised learning, and then pseudo-labeling techniques can be employed to generate pseudo-labels for unannotated data, iteratively updating the model to learn effective information from the unlabeled data, thereby enhancing segmentation performance. In the context of unsupervised learning, autoencoder networks can be constructed to input medical images, allowing the network to learn the intrinsic feature representations of the data. During the decoding phase, the original image is reconstructed, which in turn improves its performance and reliability in real-world medical image annotation tasks.

#### 6.3.2. Exploration of Lightweight and Efficient Models

In the field of medical image segmentation, complex models such as Transformers have achieved significant breakthroughs in precise segmentation due to their powerful feature extraction and representation capabilities. However, these models face limitations in clinical deployment due to their high computational costs. Additionally, their relatively long inference times are not aligned with the needs of rapid diagnosis and real-time processing in clinical settings. Given these challenges, future research in medical image segmentation models will inevitably focus on lightweight design and computational optimization.

In terms of lightweight design [155], deeper exploration can be conducted at the architectural level, combining graph neural networks (GNNs) [156] to enhance structural understanding while simplifying network structures and eliminating redundant layers and parameters to make the model more compact and efficient. Computational optimization can be achieved by leveraging hardware acceleration technologies, such as GPUs, FPGAs, and Jetson platforms, which can significantly improve model performance. Additionally, advancements in electrohydrodynamic fluid-driven systems, such as valveless water pumps, offer new paradigms for efficient fluid control, which may inspire novel approaches for hardware-based medical image processing optimizations [157,158].The combined application of these strategies holds the potential to develop medical image segmentation models that meet clinical needs while being both efficient and practical.

What is more, emerging techniques such as multi-modal transformer architectures hold great promise for addressing these challenges. Multi-modal transformers can combine information from different imaging modalities (e.g., CT, MRI, and PET) to enhance feature extraction and improve the accuracy of segmentation. By integrating data from multiple sources, these models can leverage the complementary strengths of each modality to better capture complex patterns in medical images. As the use of multi-modal data continues to grow, multi-modal transformers will likely play a pivotal role in advancing the accuracy and robustness of medical image segmentation.

#### 6.3.3. Enhancing Interpretability and Clinical Trustworthiness

Although many advanced segmentation models demonstrate the excellent accuracy of segmentation, their lack of transparency in decision-making processes makes them difficult for clinicians and patients to understand, significantly affecting the clinical trustworthiness of these models. To improve model interpretability and clinical reliability, there is a strong need for the deep integration of interpretability techniques and uncertainty assessment. On the one hand, methods such as feature visualization and decision rule extraction can present the model’s internal operations in a clear and comprehensible manner, aiding clinicians in understanding the segmentation results. On the other hand, uncertainty assessment quantifies the reliability of the model’s predictions, providing clinicians with confidence intervals for the results. The combination of these approaches enables clinicians to not only understand the rationale behind the model’s decisions but also to assess the reliability of the outcomes, thereby increasing their confidence in using the model for clinical diagnosis and ensuring the effective application of medical image segmentation models in clinical practice.

#### 6.3.4. Collaborative Development of Federated Learning and Privacy Protection

In the medical field, data holds significant research and clinical value, but the high privacy sensitivity of medical data imposes strict restrictions on data sharing. Therefore, the future of medical image segmentation should focus on the integration and development of federated learning and privacy-preserving computation techniques. On one hand, federated learning has emerged as a promising solution to this challenge by enabling decentralized model training across multiple institutions without the need for data sharing. This approach allows for privacy preservation while still improving model performance. Recent advancements in privacy-preserving technologies, such as secure aggregation, differential privacy, and encryption methods, further enhance the security of data during model training and inference. These developments are critical to ensuring that medical data can be utilized for collaborative research while maintaining patient confidentiality. On the other hand, sensitive data can be protected during model training by introducing noise or using encryption mechanisms, preventing data from being stolen or tampered with during transmission or storage. The collaborative application of these technologies allows for the optimal utilization of medical data while ensuring the safeguarding of patient privacy.

## 7. Conclusions

Medical image segmentation, as a bridge between computer vision and clinical medicine, plays an irreplaceable role in disease diagnosis, surgical planning, and treatment evaluation. Today, medical image analysis across various fields leverages artificial intelligence in one way or another, whether it involves routine X-ray examinations [159], SPECT [160], CT [161], MRI [161], or invasive coronary angiography [162].The introduction of deep learning techniques, particularly the innovations of convolutional neural networks and Transformers, has significantly enhanced the segmentation accuracy and automation, driving the transition of medical image analysis from traditional handcrafted features to data-driven methods. Despite significant progress, challenges remain in the clinical application of deep learning methods for medical image segmentation. Key limitations include data scarcity, domain shifts, overfitting, and the high computational costs associated with complex models. While current techniques exhibit promising performance in controlled environments, their robustness in real-world clinical settings is still an area that requires substantial improvement.

Looking ahead, future research should prioritize enhancing model generalization, mitigating data inefficiencies, and improving the interpretability of deep learning models to accelerate their clinical adoption. Advances in semi-supervised and unsupervised learning, coupled with more efficient network architectures, present promising avenues to address several of these challenges. Additionally, the integration of multi-modal and multi-task learning techniques could further enhance segmentation performance across varied clinical scenarios.

In conclusion, while deep learning has fundamentally transformed medical image segmentation, ongoing research is critical to overcome its current limitations and broaden its clinical applicability. As these methods continue to mature, they have the potential to substantially improve patient outcomes, ultimately advancing personalized and more effective healthcare systems.

## Figures and Tables

**Table 1:** Comparison of polyp segmentation methods performance [30].

Datasets	Methods	Rec	Spec	Prec	Dice	MAE	*S* *_**α**_*	*E*_ϕ_
Kvasir-SEG	UNet	87.89	97.96	83.89	82.85	n/a	n/a	n/a
	U-Net++	88.67	97.49	83.17	82.80	n/a	n/a	n/a
	ACSNet	93.14	91.59	97.64	91.30	3.70	89.30	92.80
	PraNet	91.41	89.56	97.25	90.75	2.90	88.20	90.80
	SANet	93.24	91.55	96.58	91.57	3.80	89.30	92.10
	ICGNet	93.70	98.31	92.63	92.35	2.70	93.15	96.24
	VANet	-	-	-	-	2.50	92.30	96.10
	UMNet	94.65	92.81	97.87	93.04	2.31	93.82	96.66
EndoScene	UNet	85.54	98.75	83.56	80.31	n/a	n/a	n/a
	U-Net++	5978.90	99.15	86.17	77.38	n/a	n/a	n/a
	ACSNet	87.96	99.16	90.99	86.59	2.84	90.45	94.07
	PraNet	82.94	99.03	90.52	83.34	2.31	90.39	92.91
	SANet	89.63	-	90.34	87.32	1.97	92.11	94.24
	ICGNet	88.45	88.45	91.24	87.93	1.89	92.42	95.04
	VANet	-	-	-	-	-	-	-
	UMNet	91.29	-	90.19	89.26	1.38	93.14	95.81

**Table 2:** Comparison of segmentation performance of polyps with typical models based on TransFormer and U-Net [56].

Datasets	ClinicDB		Kvasir		EndoScene	
Metrics	IoU	Dice	IoU	Dice	IoU	Dice
U-Net	84.32	89.28	77.58	82.31	75.23	84.36
AttU-Net	84.24	89.51	76.62	81.95	75.07	84.98
U-Net++	83.33	88.94	80.05	84.16	77.31	86.31
Deeplabv3+	84.75	90.33	81.67	85.70	72.88	84.14
nnU-Net	84.43	89.77	84.33	87.27	76.05	85.09
Trans-Unet	84.98	90.30	83.34	86.64	73.44	84.63
Swin-Unet	83.78	89.47	83.65	87.50	75.56	85.30
$I^{2}$U-Net	87.60	92.32	84.45	87.75	77.87	87.41

**Table 3:** Comparison of segmentation performance of typical methods based on TransFormer and U-Net architectures for brain tumor and spleen segmentation tasks [62].WT, ET, and TC represent the overall tumor, enhanced tumor, and tumor core subregion, respectively.

Task/Modality	Spleen Segmentation (CT)				Brain Tumor Segmentation (MRI)					
			WT		ET		TC		All	
Metrics	Dice	HD95	Dice	HD95	Dice	HD95	Dice	HD95	Dice	HD95
UNet	0.953	4.087	0.766	9.205	0.561	11.122	0.665	10.243	0.664	10.190
AttUNet	0.951	4.091	0.767	9.004	0.543	10.447	0.683	10.463	0.665	9.971
SETR NUP	0.947	4.124	0.697	14.419	0.544	11.723	0.669	15.192	0.637	13.778
SETR PUP	0.949	4.107	0.696	15.245	0.549	11.759	0.670	15.023	0.638	14.009
SETR MLA	0.950	4.091	0.698	15.503	0.554	10.237	0.665	14.716	0.639	13.485
TransUNet	0.950	4.031	0.706	14.027	0.542	10.421	0.684	14.501	0.644	12.983
TransBTS	-	-	0.779	10.030	0.574	9.969	0.735	8.950	0.696	9.650
CoTr w/ oCNN encoder	0.946	4.748	0.712	11.492	0.523	9.592	0.698	12.581	0.6444	11.221
CoTr	0.954	3.860	0.746	9.198	0.557	9.447	0.748	10.445	0.683	9.697
UNETR	0.964	1.333	0.789	8.266	0.585	9.354	0.761	8.845	0.711	8.822

**Table 4:** Performance comparison of supervised medical image segmentation approaches.

Method	Core Features	Advantages	Limitations	Applicable Scenarios
RITM	Achieves high-quality image segmentation without prior mask information	Capable of segmenting multiple complex structures across different imaging modalities	Requires significant computational resources and time	Multi-modal brain image structure segmentation
S2VNet	Achieves continuous prediction by compressing target information to centroids and passing it between adjacent slices	Achieves volumetric image segmentation using only a 2D network and can handle multiple categories simultaneously	Only handles multi-class interactions of the same category	Volumetric image segmentation of multiple targets within the same class, such as lung nodule segmentation
VANet	Introduces self-attention mechanisms and CVT architecture	Enhances feature representation of polyps	Struggles to distinguish polyps from other tissues, prone to misclassification	Colonoscopic polyp segmentation, where boundary accuracy is not extremely critical
ICGNet	RCG addresses low-contrast boundaries and missed detection issues; ALGM provides a larger acceptable range	Improves segmentation performance	Ignores inconsistencies in image color distribution, leading to overfitting and difficulty focusing on valuable image content	Boundary detection and feature fusion required, with relatively consistent color distribution, such as in normal tissue boundary segmentation
UM-Net	Introduces color transfer operations to weaken the relationship between color and polyps, making the model focus on shape	Addresses issues like inconsistent color distribution, low contrast, and misdiagnosis	Requires further model design and training improvements for more complex scenarios, such as handling background brightness variations	Polyp segmentation with inconsistent color distribution but relatively stable structure, such as under varying lighting conditions
AVDNet	Proposes two distinct types of neural networks: image feature recognition network and topology optimization network	Enables segmentation of both coronary arteries and veins with high accuracy and reliability	Currently limited to coronary artery and vein segmentation, with performance in other vascular types yet to be validated	Coronary artery and vein segmentation scenarios
Attention U-Net	Introduces attention modules on the classic U-Net architecture to guide the model’s focus on target region features	Improves segmentation accuracy and model robustness, enhancing decision interpretability	Relies on high-quality annotated data, lacks global context information mining	Scenarios requiring high accuracy in target region segmentation, with sufficient hardware support and high-quality labeled data, such as tumor segmentation
U-Net++	Uses nested skip connections on top of U-Net to fully integrate features from different depths, enhancing feature expression	Strengthens the model’s ability to capture subtle structures and boundary information in medical images	Long training time and high hardware resource requirements	Scenarios with high demand for fine structure and boundary segmentation in medical images, such as fine segmentation of neural images
R2U-Net	Incorporates recurrent structures and residual blocks into U-Net, using the recurrent structure to capture temporal information and residual blocks to mitigate vanishing gradients	Better handles medical images with complex textures and contextual information	Recurrent structure increases computational complexity and training time; improper design may make the model more sensitive to noise	Segmentation of medical images with complex textures and contextual information, such as liver regions with intricate textures
I^2^U-Net	Enhances information interaction mechanisms to capture comprehensive features during feature extraction	Accurately identifies subtle differences between various tissues and lesions in complex textured and diverse structure medical images	Increases model design and training complexity, requiring more resources for parameter optimization to achieve optimal segmentation	Complex textured and diverse structured medical image segmentation, such as chest images containing various tissues and lesions
nnU-Net	Automatically adapts to different datasets	Can be quickly deployed and achieve good results in various medical image segmentation tasks	May not perform as well on specific datasets or complex tasks compared to manually fine-tuned models	Rapid deployment in various medical image segmentation tasks, where accuracy requirements for specific datasets and complex tasks are not extremely high
TransUNet	Introduces Transformer into medical image segmentation	Significantly improves segmentation accuracy and robustness, reducing training time and data requirements	High computational resource demand, slower inference speed	Scenarios requiring high segmentation accuracy and robustness, with some computational resources available and less emphasis on inference speed, such as fine brain image segmentation
Swin-UNet	Introduces Swin Transformer as the backbone, with a hierarchical window attention mechanism	Enhances computational efficiency while maintaining Transformer’s global modeling capability	Poor interpretability of decision-making process	Medical image segmentation scenarios requiring computational efficiency and Transformer’s global modeling capability, such as mid-sized organ segmentation
Unetr	Uses a pure Transformer architecture, directly feeding medical images as sequences into the Transformer encoder	Precisely handles high-resolution medical images and complex structures	High computational resource demand, long training and inference time	Medical image segmentation scenarios requiring computational efficiency and Transformer’s global modeling capability, such as mid-sized organ segmentation
MedFormer	Proposes a multi-scale window attention module combined with local and global context information	Accurately segments vessels of varying sizes, performing well on medical image segmentation with complex scale variations	May overlook details when handling small targets due to global attention	Medical image segmentation scenarios with complex scale variations, such as segmenting vessels of different sizes
SegAN	Introduces the adversarial training mechanism of GAN into medical image segmentation tasks	Learns complex features and distributions from medical image data	Complex training process, high computational cost, less effective on small targets or boundary details	Medical image data feature learning scenarios, where high accuracy in small target or boundary detail segmentation is not critical, such as coarse organ segmentation
cGAN	Introduces conditional information into both the generator and discriminator, allowing the generator to produce segmentation results relevant to the input image	Increases the alignment of generated results with actual needs	Highly dependent on the quality and selection of conditional information	Scenarios requiring high alignment of generated results with specific conditions, such as lesion segmentation based on specific conditions
pix2pix	Based on conditional GAN, implements precise mapping from input image to target image by introducing conditional inputs	Generates high-quality images with excellent visual effects, maintaining image structure and semantic information	Requires large amounts of paired labeled data for training, high labeling cost, and relatively complex model architecture	Suitable for image-to-image translation tasks

**Table 5:** Performance comparison of consistency regularization methods in heart segmentation tasks [94].

Methods	Scans Used		Metrics			
	Labeled	Unlabeled	Dice	Jaccard	95HD	ASD
UA-MT			82.26	70.98	13.71	3.82
SASSNet			81.6	69.63	16.16	3.58
DTC			81.25	69.33	14.9	3.99
URPC	4 (5%)	76 (95%)	82.48	71.35	14.65	3.65
MC-Net			83.59	72.36	14.07	2.7
SS-Net			86.33	76.15	9.97	2.31
BCP			88.02	78.72	7.9	2.15
UA-MT			87.79	78.39	8.68	2.12
SASSNet			87.54	78.05	9.84	2.59
DTC			87.51	78.17	8.23	2.36
URPC	8 (10%)	72 (90%)	86.92	77.03	11.13	2.28
MC-Net			87.62	78.25	10.03	1.82
SS-Net			88.55	79.62	7.49	1.9
BCP			89.62	81.31	6.81	1.76

**Table 6:** Performance comparison of semi-supervised medical image segmentation algorithms.

Method	Core Features	Advantages	Limitations	Applicable Scenarios
Pseudo-Labeling with Confidence Thresholding	Uses confidence thresholding to filter out noise	Reduces the interference of incorrect labels in model training, allowing more effective use of unlabeled data	High confidence thresholds may lead to an imbalanced class distribution in pseudo-labels	Semi-supervised classification of common medical images with broad disease categories
Curriculum Semi-Supervised Learning	Introduces additional constraints to enhance pseudo-label confidence	Effectively prevents the accumulation of training errors due to incorrect pseudo-labels	Longer training times	Semi-supervised medical image segmentation where pseudo-label accuracy is critical and sufficient training time is available
CCSM	Uses a confidence calculation module to generate pseudo-labels	Generates more reliable pseudo-labels	Complex model structure, sensitive to parameters and hyperparameters	High accuracy cardiac structure segmentation tasks
SC-SSL	Improves learning confidence of unlabeled data via self-correction modules	Effectively reduces noise in pseudo-labels	Performance is highly dependent on data quality	Semi-supervised medical image segmentation scenarios with high data quality
DAN	Adaptive noise label correction	Improves pseudo-label quality	Sensitive to the choice of data transformation methods	Semi-supervised medical image segmentation requiring high pseudo-label quality
BCP	Proposes a bidirectional copy–paste method to address label distribution imbalance in semi-supervised medical image segmentation	Utilizes unlabeled data to improve model performance	Difficulty in determining suitable copy–paste regions	Semi-supervised medical image segmentation with significant label data distribution imbalance
ARCO	Proposes a group sampling-based semi-supervised learning framework	Improves model performance, reduces training time	Requires manual selection of group sampling strategies	Semi-supervised scenarios with limited labeled data
Π-model	Applies the same or different dropout perturbations to the same input	Enhances model generalization capabilities	Difficulty in determining appropriate hyperparameters and consistency loss weight during training	Semi-supervised scenarios with limited labeled data
Temporal Ensembling	Uses an exponential moving average of historical predictions as a consistency target to constrain current predictions	Reduces reliance on labeled data to improve model performance	Requires storing predictions from multiple time steps, increasing memory overhead	Semi-supervised scenarios with limited labeled data
CCT	Applies consistency constraints to model predictions under different perturbations	Enhances model performance by leveraging unlabeled data and can be extended to other weakly supervised tasks	May lead to overfitting in cases of imbalanced data distributions	Tasks requiring a large amount of unlabeled data to enhance model performance
FixMatch	Applies varying intensities of data augmentation to the same unlabeled sample	Reduces the risk of incorrect label propagation	Sensitive to hyperparameter settings	Tasks requiring large amounts of unlabeled data to improve model performance
Mean Teacher	Uses the average model weights as targets to improve semi-supervised learning effectiveness	Improves test accuracy, trains with fewer labeled data, and does not require changes to network architecture	Targets generated by the teacher model may contain noise and unreliability	Tasks requiring large amounts of unlabeled data to improve model performance
UA-MT	Proposes an uncertainty-aware self-supervised learning framework	Effectively utilizes unlabeled data to improve segmentation accuracy	May overfit with limited data availability	Tasks requiring large amounts of unlabeled data to improve model performance
CCT	Enforces consistency of perturbations on the encoder’s output	Improves the encoder’s representational ability	Requires significant computational resources for training	Tasks lacking large labeled data
SS-NET	Considers pixel-level smoothness and class-level separability simultaneously	Effectively utilizes unlabeled data for semi-supervised learning, improving model performance	Requires manual setting of some hyperparameters	Scenarios with difficult data annotation
ACTION++	Proposes adaptive supervised contrastive loss	Effectively addresses the long-tail distribution and class imbalance in medical image data	High model complexity, poor interpretability	Scenarios requiring extremely high result accuracy

**Table 7:** Performance comparison of unsupervised medical image segmentation methods.

Method	Core Features	Advantages	Limitations	Applicable Scenarios
SIFA	Adaptively learns from both image and feature perspectives for cross-modal medical image segmentation tasks	Offers good generalizability and scalability	Requires large computational resources to train the model, and may have limitations for certain specific application scenarios	Cross-modal image segmentation tasks in the medical field
DSAN	Implements bidirectional alignment of source/target domain feature distributions via shared encoders and private decoders	Fully leverages information from images with different styles	Requires significant computational resources to train the model	Cross-modal image segmentation tasks in the medical field
DSFN	Achieves collaborative alignment of source and target domains from both image-level and feature-level perspectives	Effectively narrows domain gaps and utilizes task complementarity	Requires significant computational resources to train the model	Medical image segmentation scenarios with domain shift challenges, such as brain tumor and heart structure segmentation
SIDA	Introduces a baseline model combining image and feature alignment, innovatively adding image translation degree prediction and contrastive learning self-supervised tasks	Effectively enhances domain adaptation performance	Not well adapted to cases with large data distribution differences	Unsupervised domain adaptation tasks in medical image segmentation
FDA	Reduces differences between source and target images by exchanging low-frequency information without any training process	Simple, intuitive, and highly efficient	Cannot handle high-frequency information, potentially losing some detailed information	Scenarios with significant differences between source and target datasets
FIESTA	Uses a Fourier-domain adaptation approach combined with uncertainty-guided data augmentation to enhance model generalization	Effectively handles detail and uncertainty issues	Limited to single-source domain generalization, may not perform well for multi-source domains	Single-source dataset tasks
DAG-Net	Proposes FCSA and RSA modules based on Fourier transform to achieve efficient cross-modal domain adaptation	Outperforms existing domain adaptation methods in cross-modal transfer tasks	Requires high computational resources and longer training times	Cross-modal transfer tasks in 3D medical image segmentation
MAPSeg	Proposes a joint learning framework based on 3D mask autoencoders, global–local context, and large-scale pre-training	Capable of handling various domain adaptation tasks, enhancing model generalization	Requires a large amount of labeled data for pre-training	Medical image segmentation tasks requiring handling of multi-source heterogeneous data
ProCA	Combines prototype contrastive learning and domain adaptation for unsupervised domain adaptation	No target domain labels required, enhances feature discriminability, significantly improves performance on the target domain	Relies on the quality of source domain labels, pseudo-label noise may affect prototype computation accuracy	Unsupervised domain adaptation tasks such as cross-domain image classification and semantic segmentation
CLMorph	Combines contrastive learning with image registration	Highly versatile, applicable to multiple medical image modalities	Dependent on registration accuracy when handling complex anatomical structures	Segmentation of CT, MRI, and other modalities in scenarios with scarce labeled data
MLIP	Combines medical domain expertise with contrastive learning to enhance medical visual representation	Improves model generalization capabilities	Relies on medical domain expertise	Medical image classification, object detection, and semantic segmentation tasks
MedSAM	Introduces SAM into the field of medical image segmentation for the first time	High generalizability and flexibility	Requires reliance on medical domain expertise	Accurate and rapid localization and segmentation of various tissues, organs, or lesion areas

**Table 8:** Common medical image datasets.

Part	Imaging Modality	Name	Size	Format	Area	Address
Abdominal Organ	CT	BTCV [133]	50	NIFIT	Spleen, right kidney, left kidney, gallbladder, esophagus, liver, stomach, aorta, inferior vena cava, portal and splenic veins, pancreas, right adrenal gland, left adrenal gland	https://aistudio.baidu.com/datasetdetail/107078 (accessed on 27 April 2025)
	CT	AMOS [134]	600	NIFIT	Spleen, right kidney, left kidney, gallbladder, esophagus, liver, stomach, aorta, inferior vena cava, pancreas, right adrenal gland, left adrenal gland, duodenum, bladder, prostate/uterus	https://zenodo.org/records/7155725#.Y0OOCOxBztM (accessed on 27 April 2025)
	CT	NIH Pancreas-CT [135]	82	NIFIT	Pancreatic	https://www.cancerimagingarchive.net/collection/pancreas-ct/ (accessed on 27 April 2025)
	CT	Task07_Pancreas [136]	420	NIFIT	Pancreas, Pancreatic tumors	https://pan.baidu.com/s/1fNRLPJuwGQWbwquSfrM1pw?pwd=2024 (accessed on 27 April 2025)
	Endoscopy	CVC-ClinicDB [137]	612	PNG	Colorectal	https://aistudio.baidu.com/datasetdetail/65816/1 (accessed on 27 April 2025)
	Endoscopy	Kvasir-SEG [138]	1000	JPG	Colon	https://datasets.simula.no/downloads/kvasir-seg.zip (accessed on 27 April 2025)
	Endoscopy	EndoScene [30]	912	JPEG, PNG	Colon	-
Chests	MRI	ACDC [139]	150	NIFIT	Heart	https://aistudio.baidu.com/datasetdetail/267540 (accessed on 27 April 2025)
	MRI	LA [104]	154	nrrd	Left atrium	https://www.cardiacatlas.org/atriaseg2018-challenge/atria-seg-data/ (accessed on 27 April 2025)
	CT MRI	MM-WHS [140]	120	NIFIT	Seven cardiac substructures	https://mega.nz/folder/UNMF2YYI#1cqJVzo4p_wESv9P_pc8uA (accessed on 27 April 2025)
	Chest X-ray	JSRT [141]	247	PNG	Lung	http://db.jsrt.or.jp/eng.php (accessed on 27 April 2025)
	Chest X-ray	ChestX-ray14 [142]	112,120	PNG	Lung, Heart	https://aistudio.baidu.com/aistudio/data (accessed on 27 April 2025)
	Chest X-ray	LUNA16 [143]	888	mhd	Lung/lung nodules	https://luna16.grand-challenge.org/Download/ (accessed on 27 April 2025)
	CT	SegTHOR [144]	60	NIFIT	Heart, Trachea, Aorta, Esophagus	https://competitions.codalab.org/competitions/21145#participate-get_starting_kit (accessed on 27 April 2025)
Brain	MRI	BraTs2018 [145]	285	NIFIT	Glioma	https://aistudio.baidu.com/aistudio/datasetdetail/64660 (accessed on 27 April 2025)
	MRI	Mindboggle [146]	101	NIFIT	Brain structure	https://mindboggle.info/data.html (accessed on 27 April 2025)
Eye	Color Fundus Photography	DRIVE [147]	40	TIFF	Retinal vessels	https://gitee.com/zongfang/retina-unet/tree/master/DRIVE (accessed on 27 April 2025)
	Color Fundus Photography	REFUGE [148]	1200	JPEG	Optic disc and Optic cup	https://refuge.grand-challenge.org/ (accessed on 27 April 2025)
	Color Fundus Photography	IDRiD [149]	516	JPG	Areas of lesions associated with diabetic retinopathy	https://idrid.grand-challenge.org/Data_Download/ (accessed on 27 April 2025)
	Color Fundus Photography	CHASE_DB1 [150]	1200	JPEG	Pathological myopia Vascular lesions	https://blogs.kingston.ac.uk/retinal/chasedb1/ (accessed on 27 April 2025)
Kidney	CT	KiTS19 [151]	300	NIFIT	Renal tumor	https://github.com/neheller/kits19 (accessed on 27 April 2025)
	CT MRI	TCIA [152]	-	DICOM	Renal parenchyma, renal cysts, renal tumors, etc.	http://www.cancerimagingarchive.net/ (accessed on 27 April 2025)
Pancreas	CT	3D-IRCADb [153]	22	DICOM	Liver, liver vessels	https://aistudio.baidu.com/datasetdetail/107717 (accessed on 27 April 2025)
